# Predictive accuracy of diagnostic tests for excessive bleeding in cardiac surgery: The COPTIC‐C study

**DOI:** 10.1111/trf.18399

**Published:** 2025-10-19

**Authors:** Weiqi Liao, Robert Grant, Florence Y. Lai, Hardeep Aujla, Marcin Wozniak, Hasmukh R. Patel, Laura Green, Andrew Mumford, Gavin J. Murphy

**Affiliations:** ^1^ Department of Cardiovascular Sciences University of Leicester UK; ^2^ Blizard Institute Queen Mary University of London UK; ^3^ NHS Blood and Transplant London UK; ^4^ Bristol Heart Institute University of Bristol UK

**Keywords:** biomarkers, cardiac surgery, diagnostic tests, postoperative excessive bleeding, predictive accuracy

## Abstract

**Background:**

We tested the hypothesis that the addition of biomarkers of multimorbidity and biological aging would improve the predictive accuracy of point‐of‐care viscoelastometry or laboratory tests of coagulation for clinically important bleeding following cardiac surgery.

**Study design and methods:**

This predictive accuracy study included 2437 participants in the coagulation and platelet laboratory testing in cardiac surgery (COPTIC study) with complete clinical, TEG 5000 thromboelastography, ROTEM, multiplate aggregometry, full blood count, laboratory reference tests of coagulopathy, and biomarkers of biological aging and multimorbidity. Models with different biomarkers to predict the composite primary outcome, clinically important bleeding, were developed using logistic regression and internally validated using 10‐fold cross‐validation. Discrimination, calibration, and clinical utility of the models were assessed comprehensively.

**Results:**

For the composite primary outcome, the AUROC for the best predictive model using TEG or ROTEM plus other biomarkers was 0.694 (0.612–0.775). The best predictive model overall included laboratory reference tests of coagulation, full blood count results, and biomarkers of multimorbidity and aging, AUROC = 0.701 (0.620–0.781), although clinical utility was not superior to using laboratory reference tests alone. Discrimination was higher for individual components of the primary outcome: large volume (≥4 units) red cell transfusion 0.754 (0.602–0.903) and large volume procoagulant transfusion 0.723 (0.590–0.857), but not for excess loss in drains/re‐sternotomy 0.701 (0.613–0.788). Calibration was generally good among the models.

**Discussion:**

The addition of biomarkers of multimorbidity and biological aging yielded only small improvements in model predictive accuracy for bleeding over tests of coagulation. Existing clinical definitions of bleeding likely represent heterogeneous phenotypes and disease mechanisms.

**Abbreviations**
AUROC: area under the receiver operating characteristic curve
CCB: clinical concern about bleeding
CIB: clinically important bleeding

## 1 INTRODUCTION

Excessive bleeding is common following cardiac surgery where it increases the risks of postoperative infection, organ injury, and death.^1^, ^2^ Clinical interventions targeting excessive bleeding do not improve clinical outcomes beyond reductions in bleeding and transfusion requirements.^3^, ^4^ The imprecision of existing clinical definitions of bleeding and diagnostic tests is a barrier to research addressing this paradox.

Consensus definitions of bleeding focus on clinical parameters such as the volume of transfusion or blood loss, or the requirement for reintervention^1^, ^2^, ^5^, ^6^, ^7^ rather than disease mechanisms, which are poorly understood. In the coagulation and platelet laboratory testing in cardiac surgery (COPTIC) study (ISRCTN20778544)^6^, ^8^ and a subsequent meta‐analysis of COPTIC and similar studies,^7^ we demonstrated that laboratory reference tests of coagulation were poor predictors of bleeding, as were point‐of‐care tests of coagulopathy based on viscoelastic tests (i.e., thromboelastography [TEG] and rotational thromboelastometry [ROTEM]), or platelet aggregation. These observations led us to hypothesize that alternative disease mechanisms that cause bleeding were unmeasured in these analyses. Chronic conditions and multimorbidity are strongly associated with bleeding in clinical studies and bleeding risk prediction scores.^9^ In a recent secondary analysis of COPTIC data, we demonstrated that biomarkers of chronic conditions and biological aging, a mechanism common to many chronic diseases, were significantly associated with the study primary outcome, clinical concern about bleeding (CCB).^10^

To improve our understanding of bleeding phenotypes, this study sought to test the hypothesis that the predictive accuracy of TEG/ROTEM or laboratory reference tests of coagulopathy would be improved by the addition of baseline biomarkers of multimorbidity or biological aging. A secondary hypothesis evaluated whether the clinical definition of the bleeding outcome influenced the results.

## 2 STUDY DESIGN AND METHODS

### 2.1 Data source

The predictive accuracy study design minimized the risk of bias for the domains identified in QUADAS‐2.^11^ The protocol was prospectively registered (ISRCTN20630689), and was reported following the STARD guidelines.^12^ The development of the predictive models was reported following the TRIPOD statement^13^ (both checklists in Appendix S2). South Central—Berkshire Research Ethics Committee approved this study (ref.: 21/SC/0118) on March 29, 2021.

Patients recruited to the COPTIC study between March 2010 and September 2012 who provided written informed consent for secondary analyses were eligible. The COPTIC clinical, TEG, ROTEM, Multiplate, Sysmex, and laboratory tests of coagulopathy datasets were linked to biological aging and organ dysfunction biomarkers by unique participant ID. Thirty‐eight participants without available lab results were excluded (eTable 1 in Supporting Information).

### 2.2 Index tests

The test data included (1) post protamine point‐of‐care TEG (TEG 5000 Thromboelastograph, Haemonetics, Coventry, UK) and INTEM, EXTEM, FIBTEM, and HEPTEM tests using ROTEM (Werfen, Cheshire, UK) in whole blood; (2) post protamine laboratory tests of coagulation; PT, APTT, Clauss fibrinogen activity, vWF Ristocetin cofactor activity, FXIII activity, D‐dimer concentration, anti‐Xa assay using an unfractionated heparin standard, and thrombin generation. (3) Post protamine platelet aggregometry in whole blood (ADP‐test, ASPI‐test, TRAP‐test, Adren‐test) used the multiplate device; (4) post protamine full blood count (FBC) was measured using the Sysmex X2100 Analyser, all performed at the time of the primary study, plus (5) pre‐surgery biomarkers of multimorbidity (organ dysfunction); serum bilirubin, alkaline phosphatase, full blood count, glomerular filtration rate (eGFR) estimated from serum creatinine, transferrin, ferritin, interleukins (IL)‐6, IL‐8, high sensitivity serum Troponin I, and pro‐NT BNP and (6) biomarkers of biological aging including CCL11/Eotaxin, CX3CL1/Fractalkine, GDF‐15, IL5, and CXCL9/iAge, all measured in thawed from frozen, double spun plasma collected during the primary study and stored at −80°C until batch analyses. The methods for these assays have been described in detail previously.^6^, ^10^

### 2.3 Clinical outcomes

The pre‐specified primary outcome was clinically important bleeding (CIB), defined as ANY of the following events:

1. *Large volume red cell transfusion* (≥4 units) from the administration of protamine to 24 h post‐surgery;
2. *Large volume transfusion of pro‐coagulants* or clotting factors was defined based on our clinical experience of treating clinically important bleeding: FFP transfusion >2 units, cryoprecipitate transfusion >2 units, platelet transfusion >1 unit, or use of recombinant Factor VII from the administration of protamine to 24 h post‐surgery.
3. *Severe blood loss* was defined as loss in drains >600 mL in 4 h post return to ICU, or emergency re‐sternotomy for bleeding, where a ‘surgical’ cause had been excluded.

The secondary outcomes included

- Individual components of the primary outcome (above),
- Postoperative Clinical Concern About Bleeding (Postop CCB) as defined in the original COPTIC study,^6^
- A composite clinical outcome of in‐hospital death, KDIGO‐defined Stage 2 or Stage 3 acute kidney injury, myocardial infarction, stroke, and infection, occurring within the index hospitalization, all as defined in the primary publication.^6^

### 2.4 Participant flow and clinical management

**FIGURE 1:**
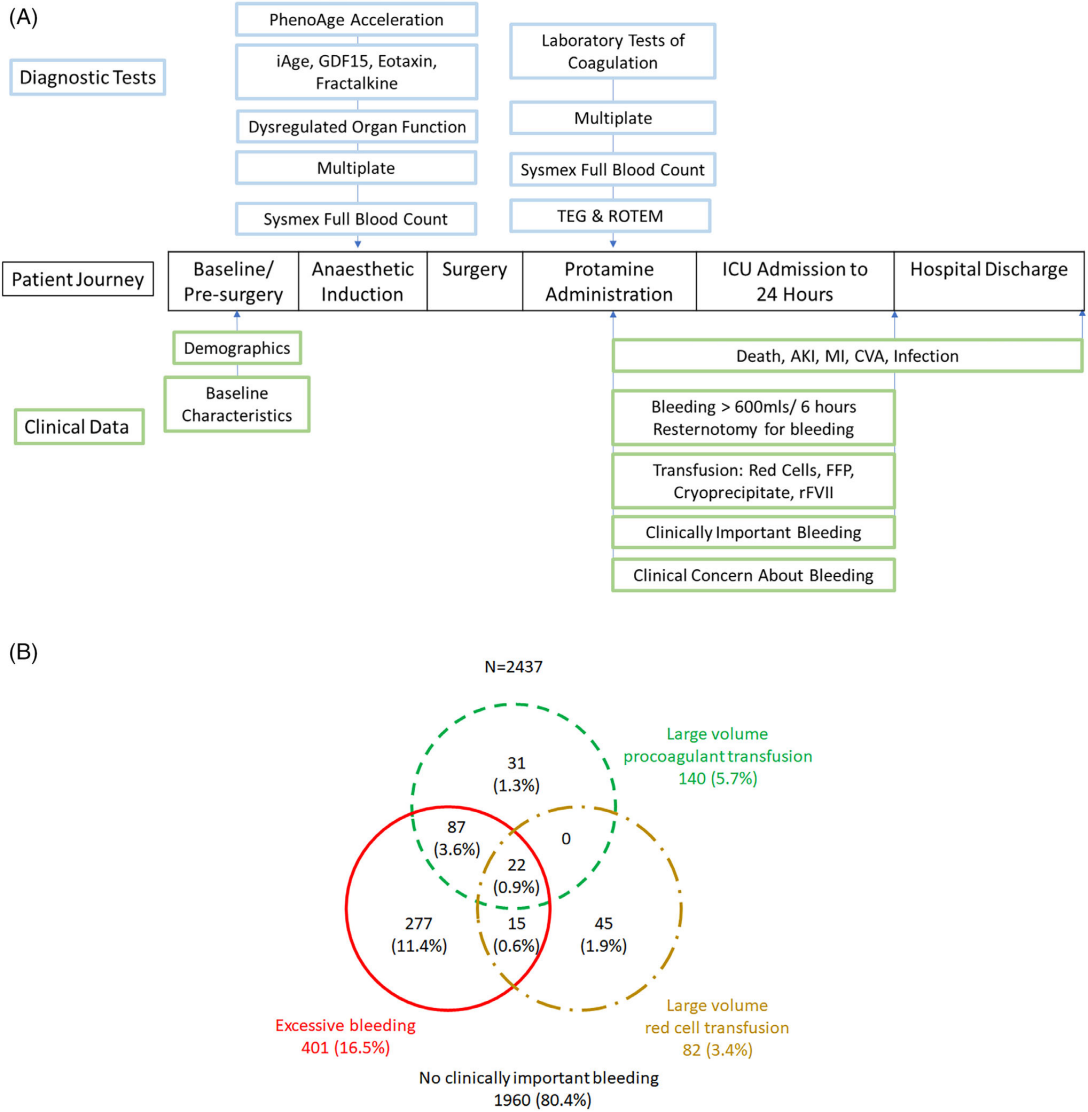
Flowchart and diagram of the study. (A) Flowchart of the patient journey, the timings of diagnostic tests, and the primary and secondary clinical outcomes. (B) Venn diagram of the three components for clinically important bleeding.

A schematic of participant flow through the study is shown in Figure 1. Two 22.5 mL blood samples (45 mL in total) were obtained in the operating theater for research at the following time points:

- Immediately before induction of anesthesia (pre‐surgery),
- Twenty minutes post‐reversal of heparin anticoagulation (post‐protamine, before chest closure).

Assessment of the primary outcome was restricted to after the routine administration of protamine before chest closure, and within 24 hours of admission to the intensive care unit. Assessment of secondary outcomes was from protamine administration until hospital discharge or death.

Decisions about intra‐ and post‐operative hemostasis and transfusion treatment were guided by existing unit protocols and ad hoc TEG analyses performed without knowledge of the TEG/ROTEM/multiplate measured remotely and standard laboratory screening tests of coagulation (PT, APTT, ACT, fibrinogen, platelet count) performed at the discretion of the responsible clinician.

### 2.5 Statistical analyses

We checked for outliers and missing values before any statistical analyses to ensure data quality. Normalization or transformation of the biomarkers was conducted when necessary. We reported descriptive statistics by patients with and without clinically important bleeding, using mean and standard deviation (SD) or quartiles (median, Q1, Q3) as appropriate for continuous variables, and frequency (*n*) and percentage (%) for binary and categorical variables. Multiple imputation with chained equations was used to impute missing values for laboratory results using predictive mean matching for continuous variables with 10 imputed datasets. Results were combined using Rubin's rules.^14^ We investigated the association between individual variables and CIB using univariable logistic regression and reported the odds ratio (OR) and 95% confidence interval (95% CI), with the *p* value.

We investigated the diagnostic accuracy of individual parameters of TEG, ROTEM, pre‐surgery organ dysfunction biomarkers, biological aging biomarkers, post‐protamine laboratory tests of coagulation, multiplate aggregometry, and Sysmex for CIB and its three components and reported area under the receiver operating characteristic curve (AUROC) and 95% CI. We compared the AUROC values between the continuous and binary forms for each parameter/biomarker, where the binary form was based on pre‐specified thresholds (eTables A–D in Supporting Information) to dichotomize the continuous values into normal and abnormal test results. Where cut‐offs for biomarkers were not well established, only continuous values were used in the analyses. Sensitivity analysis was conducted when restricted to participants with clinical concern about bleeding, as defined in the primary COPTIC analysis.

Different modeling strategies were used to predict CIB and its three components. When using variables from multiple test domains, two machine learning methods, stepwise backward elimination and Lasso (least absolute shrinkage and selection operator) logistic regression, were used to guide variable selection in the model development stage in the imputed datasets, which was an iterative process. We retained variables with statistical significance (*p* < .05) in the multi‐variable models. We balanced model parsimony and clinical utility when selecting the best set of predictors for the final model. A simple model with reasonably satisfactory model performance and the potential to translate into clinical practice is preferred to complicated models with more variables. The models were then internally validated using 10‐fold cross‐validation, which is a technique to evaluate model performance by splitting the dataset randomly into 10 equal parts (folds), training the model on 9 folds, and testing the model on the remaining fold, recording the model performance for each fold. This process was repeated 10 times, each fold serving as the test set once. Finally, the performance metrics were averaged over the 10 folds to assess the model. Model performance was evaluated by AUROC and 95% CI for discrimination (whether an individual would have a positive binary outcome or not), observed‐to‐expected (O:E) Ratio and Brier score for calibration (the agreement between the observed frequencies and the predictive probabilities of the outcome), and net benefit for clinical utility (balance the true positives and false positive across probability thresholds) using decision curve analysis.^15^ AUROC values were interpreted as poor <0.7, moderate 0.7–0.8, good 0.8–0.9, and excellent >0.9.^16^, ^17^ Models with Brier scores close to 0 and O:E ratio close to 1 have better calibration. All analyses were conducted in Stata 18.

## 3 RESULTS

### 3.1 Analysis cohort and the prevalence of primary and secondary outcomes

COPTIC participants included (*n* = 2437) and excluded (*n* = 38, 1.5%) from this study were similar for all baseline clinical variables except for age, which was younger in the excluded cohort (eTable 1 in Supporting Information). In the analysis population, participants who developed clinically important bleeding 477/2437 (19.6%) were more likely to be older (68.8 ± 11.6 years vs. 66.2 ± 11.9 years), male (81.6% vs. 73.8%), have lower BMI (26.9 ± 4.8 vs. 28.2 ± 4.7), and moderate or poor left ventricular function, anemia (47.9% vs. 36.7%), peripheral vascular disease (12.4% vs. 8.7%), had a higher percentage of valve surgery (49.4% vs. 39.4%), combined CABG and valve surgery (21.5% vs. 11.1%), and aortic surgery (7.7% vs. 4.4%), and longer cardiopulmonary bypass times (average 86.93 min vs. 60.88 min) and higher EuroScore (5.84 ± 3.17 vs. 4.76 ± 2.84) (all statistically significant, Table 1), compared with those who did not. Clinically important bleeding increased the likelihood of developing a composite secondary outcome of MI, stroke, Stage 3 AKI, sepsis, or death; OR = 2.3 (95%CI 1.85–2.92).

**TABLE 1:** Baseline clinical characteristics in participants with and without clinically important bleeding.

	Clinically important bleeding				
	No	Yes	Total	Crude odds ratio (95% CI)	*p* value
*N* (%)	1960 (80.4%)	477 (19.6%)	2437 (100.0%)		
Age at surgery	66.24 (11.85)	68.80 (11.59)	66.74 (11.84)	1.020 (1.011–1.030)	<.001
Sex					
Male	1447 (73.8%)	389 (81.6%)	1836 (75.3%)	Reference	—
Female	513 (26.2%)	88 (18.4%)	601 (24.7%)	0.638 (0.496–0.821)	<.001
Ethnicity					
White	1846 (94.2%)	455 (95.4%)	2301 (94.4%)	Reference	—
Asian/Black/mixed	31 (1.6%)	4 (0.8%)	35 (1.4%)	0.524 (0.184–1.491)	.225
Unknown	83 (4.2%)	18 (3.8%)	101 (4.1%)	0.880 (0.523–1.480)	.629
BMI	28.21 (4.71)	26.94 (4.78)	27.96 (4.75)	0.940 (0.918–0.962)	<.001
BMI ≥ 30					
No	1297 (67.6%)	375 (79.4%)	1672 (70.0%)	Reference	—
Yes	621 (32.4%)	97 (20.6%)	718 (30.0%)	0.540 (0.424–0.689)	<.001
Smoking history					
Never smoked	746 (38.2%)	191 (40.2%)	937 (38.6%)	Reference	—
Ex‐smoker	1132 (57.9%)	267 (56.2%)	1399 (57.6%)	0.921 (0.749–1.134)	.438
Current smoker	77 (3.9%)	17 (3.6%)	94 (3.9%)	0.862 (0.498–1.493)	.597
NYHA ≥ 3					
No	1342 (68.7%)	312 (65.8%)	1654 (68.1%)	Reference	—
Yes	612 (31.3%)	162 (34.2%)	774 (31.9%)	1.139 (0.921–1.408)	.231
Previous MI (binary)					
No	1322 (68.0%)	300 (63.2%)	1622 (67.0%)	Reference	—
Yes	623 (32.0%)	175 (36.8%)	798 (33.0%)	1.238 (1.004–1.526)	.046
CCS ≥ 2					
No	870 (44.5%)	232 (48.8%)	1102 (45.4%)	Reference	—
Yes	1084 (55.5%)	243 (51.2%)	1327 (54.6%)	0.841 (0.688–1.028)	.090
Previous PCI					
No	1773 (90.9%)	434 (91.4%)	2207 (91.0%)	Reference	—
Yes	178 (9.1%)	41 (8.6%)	219 (9.0%)	0.941 (0.660–1.343)	.737
Ejection fraction category					
Good (>50%)	1558 (79.8%)	350 (73.4%)	1908 (78.6%)	Reference	—
Fair (30%–49%)	296 (15.2%)	96 (20.1%)	392 (16.1%)	1.444 (1.116–1.868)	.005
Poor (<30%)	98 (5.0%)	31 (6.5%)	129 (5.3%)	1.408 (0.925–2.143)	.110
Anemia					
No	1231 (63.3%)	247 (52.1%)	1478 (61.1%)	Reference	—
Yes	715 (36.7%)	227 (47.9%)	942 (38.9%)	1.582 (1.292–1.937)	<.001
CKD (baseline eGFR < 60)					
No	1394 (71.4%)	327 (68.6%)	1721 (70.8%)	Reference	—
Yes	559 (28.6%)	150 (31.4%)	709 (29.2%)	1.144 (0.921–1.421)	.224
Diabetes					
No	1542 (78.9%)	397 (83.6%)	1939 (79.8%)	Reference	—
Diet controlled	72 (3.7%)	15 (3.2%)	87 (3.6%)	0.809 (0.459–1.427)	.464
Oral therapy controlled	221 (11.3%)	42 (8.8%)	263 (10.8%)	0.738 (0.521–1.045)	.087
Insulin controlled	119 (6.1%)	21 (4.4%)	140 (5.8%)	0.685 (0.425–1.104)	.121
Hypertension					
No	556 (28.6%)	151 (31.7%)	707 (29.2%)	Reference	—
Yes	1389 (71.4%)	325 (68.3%)	1714 (70.8%)	0.862 (0.694–1.070)	.178
Respiratory disease					
No	1716 (87.8%)	412 (86.6%)	2128 (87.5%)	Reference	—
Yes	239 (12.2%)	64 (13.4%)	303 (12.5%)	1.115 (0.830–1.500)	.470
Cerebrovascular disease					
No	1785 (91.4%)	419 (89.1%)	2204 (91.0%)	Reference	—
Yes	167 (8.6%)	51 (10.9%)	218 (9.0%)	1.301 (0.934–1.812)	.119
History of peripheral vascular disease					
No	1784 (91.3%)	417 (87.6%)	2201 (90.5%)	Reference	—
Yes	171 (8.7%)	59 (12.4%)	230 (9.5%)	1.476 (1.078–2.022)	.015
History of neurological dysfunction					
No	1913 (97.9%)	454 (95.4%)	2367 (97.4%)	Reference	—
Yes	42 (2.1%)	22 (4.6%)	64 (2.6%)	2.207 (1.305–3.734)	.003
PhenoAge	65.91 (13.25)	68.98 (13.62)	66.51 (13.38)	1.018 (1.009–1.027)	<.001
CABG surgery					
No	367 (31.3%)	96 (30.8%)	463 (31.2%)	Reference	—
Yes	807 (68.7%)	216 (69.2%)	1023 (68.8%)	1.023 (0.781–1.341)	.868
Valve surgery					
No	711 (60.6%)	158 (50.6%)	869 (58.5%)	Reference	—
Yes	463 (39.4%)	154 (49.4%)	617 (41.5%)	1.497 (1.165–1.924)	.002
Both CABG and valve					
No	1044 (88.9%)	245 (78.5%)	1289 (86.7%)	Reference	—
Yes	130 (11.1%)	67 (21.5%)	197 (13.3%)	2.196 (1.585–3.042)	<.001
Aortic surgery					
No	1122 (95.6%)	288 (92.3%)	1410 (94.9%)	Reference	—
Yes	52 (4.4%)	24 (7.7%)	76 (5.1%)	1.798 (1.090–2.967)	.022
Cumulative bypass time (min)	60.88 (61.12)	86.93 (88.08)	65.98 (68.02)	1.006 (1.004–1.007)	<.001
EuroSCORE	4.76 (2.84)	5.84 (3.17)	4.97 (2.94)	1.129 (1.092–1.167)	<.001
Large volume red cell transfusion (≥4 units)					
No	1789 (100.0%)	340 (80.6%)	2129 (96.3%)		
Yes	0 (0.0%)	82 (19.4%)	82 (3.7%)		
Transfusion of FFP, cryoprecipitate, platelet, or use Factor VII					
No	1960 (100.0%)	337 (70.6%)	2297 (94.3%)		
Yes	0 (0.0%)	140 (29.4%)	140 (5.7%)		
Bleeding >600 mL or emergency re‐sternotomy					
No	1958 (100.0%)	76 (15.9%)	2034 (83.5%)		
Yes	0 (0.0%)	401 (84.1%)	401 (16.5%)		
Clinical concern about bleeding					
No	1607 (89.8%)	59 (13.9%)	1666 (75.3%)	Reference	—
Yes	182 (10.2%)	364 (86.1%)	546 (24.7%)	54.475 (39.759–74.637)	<.001
Composite clinical (secondary) outcomes					
No	1642 (83.8%)	329 (69.0%)	1971 (80.9%)	Reference	—
Yes	318 (16.2%)	148 (31.0%)	466 (19.1%)	2.323 (1.849–2.918)	<.001
Acute kidney injury (AKI)					
No AKI	1141 (58.7%)	204 (42.9%)	1345 (55.6%)	Reference	—
AKI Stage 1	581 (29.9%)	165 (34.7%)	746 (30.8%)	1.588 (1.264–1.996)	<.001
AKI Stage 2	121 (6.2%)	41 (8.6%)	162 (6.7%)	1.895 (1.291–2.783)	.001
AKI Stage 3	101 (5.2%)	66 (13.9%)	167 (6.9%)	3.655 (2.591–5.156)	<.001
Myocardial infarction					
No	1924 (98.6%)	467 (99.4%)	2391 (98.8%)	Reference	—
Yes	27 (1.4%)	3 (0.6%)	30 (1.2%)	0.458 (0.138–1.515)	.201
Stroke					
No	1937 (99.3%)	451 (96.0%)	2388 (98.6%)	Reference	—
Yes	14 (0.7%)	19 (4.0%)	33 (1.4%)	5.829 (2.901–11.713)	<.001
Sepsis					
No	1852 (95.0%)	408 (87.7%)	2260 (93.6%)	Reference	—
Yes	97 (5.0%)	57 (12.3%)	154 (6.4%)	2.667 (1.891–3.763)	<.001
Death					
No	1934 (99.0%)	448 (93.9%)	2382 (98.0%)	Reference	—
Yes	19 (1.0%)	29 (6.1%)	48 (2.0%)	6.589 (3.661–11.857)	<.001

*Note*: Mean (standard deviation) for continuous variables, *N* (%) for binary and categorical variables.

For the individual components of the primary outcome, large volume red cell transfusion occurred in 82/2437 (3.4%) participants, large volume/dose of procoagulants occurred in 140/2437 (5.7%), and excessive bleeding occurred in 401 (16.5%). 30.9% (124/401) patients had at least two components (Venn diagram, Figure 1B). Test results for all biomarkers in participants with and without CIB are shown in eTable 2 in Supporting Information. This univariable analysis found 35 out of 60 test parameters/biomarkers (continuous) were significantly associated with CIB (*p* < .05).

### 3.2 Diagnostic accuracy of TEG/ROTEM for laboratory diagnostic tests and aggregometry

We assessed whether TEG/ROTEM results demonstrated diagnostic accuracy for test positive thresholds of corresponding laboratory tests of coagulopathy, full blood count, and platelet function. When the threshold value for a positive test for each TEG parameter was used as binary variables in the analysis, all the AUROC values were <0.6 (eTable 3 in Supporting Information). When TEG parameters were used as continuous variables, the majority of AUROC values were <0.7, except for TEG citrate kaolin (CK) R time (rmin) for hemoglobin 0.718 (0.641–0.795) and CK heparinase rmin for anti‐Xa activity 0.852 (0.672–1.000). TEG CK maximum amplitude (MA) demonstrated moderate (AUROC < 0.8) predictive accuracy for Factor XIII, vWF, and post‐op platelet counts, and good predictive accuracy for Clauss fibrinogen 0.829 (0.812–0.847), and the product of platelet count and mean platelet volume (MPV X platelet), 0.808 (0.789–0.827).

When the threshold value for a positive test for each ROTEM parameter was used (binary variables), all the resulting AUROC values were <0.7, except for INTEM α angle and MPV X platelet, 0.703 (0.684–0.721, eTable 4 in Supporting Information). When continuous ROTEM parameters were used, the AUROC for EXTEM clotting time (CT) for PT was 0.788 (0.731–0.845), HEPTEM CT for anti‐Xa was 0.765 (0.609–0.921), INTEM *α* angle for Clauss fibrinogen was 0.848 (0.831–0.864), and 0.848 (0.832–0.864) for platelet count. FIBTEM maximum clot firmness (MCF) demonstrated AUROC 0.908 (0.895–0.920) for Clauss fibrinogen, and INTEM MCF demonstrated AUROC 0.8–0.9 for Clauss fibrinogen, platelet count, and MPV X platelet.

### 3.3 Predictive accuracy of TEG/ROTEM and laboratory tests for primary and secondary outcomes

The results of AUROC for individual TEG/ROTEM parameters for the primary outcome (CIB), three individual components of the primary outcome, and secondary outcomes in univariate analysis are in Table 2. All AUROC values were <0.7, regardless of the input of TEG/ROTEM parameters as continuous values or using the established positive test thresholds as binary variables.

**TABLE 2:** Predictive accuracy of TEG/ROTEM parameters (continuous and binary) for clinically important bleeding and its three individual components, and the secondary outcome.

Test variable	CIB_bi_AUC	CIB_con_AUC	CCB_bi_AUC	CCB_con_AUC	RBC_Transfusion_bi_AUC	RBC_Transfusion_con_AUC	ProCoagulants_bi_AUC	ProCoagulants_con_AUC	Excessive bleeding_bi_AUC	Excessive bleeding_con_AUC
TEG CK rmin	0.502 (0.493–0.510)	0.512 (0.481–0.543)	0.502 (0.494–0.509)	0.529 (0.501–0.557)	0.502 (0.484–0.519)	0.528 (0.463–0.593)	0.502 (0.490–0.515)	0.514 (0.457–0.571)	0.501 (0.492–0.510)	0.512 (0.478–0.545)
CKH rmin	^a^	0.514 (0.483–0.546)	^a^	0.518 (0.490–0.546)	^a^	0.533 (0.468–0.598)	^a^	0.524 (0.468–0.579)	^a^	0.512 (0.478–0.545)
TEG CK α angle	0.502 (0.497–0.507)	0.525 (0.493–0.556)	0.502 (0.498–0.507)	0.531 (0.503–0.559)	0.503 (0.491–0.516)	0.574 (0.505–0.642)	0.506 (0.494–0.518)	0.592 (0.539–0.645)	0.503 (0.497–0.509)	0.547 (0.514–0.580)
TEG CK MA	0.513 (0.504–0.522)	0.596 (0.566–0.627)	0.512 (0.504–0.520)	0.610 (0.582–0.638)	0.512 (0.491–0.534)	0.504 (0.433–0.575)	0.529 (0.506–0.553)	0.664 (0.613–0.716)	0.515 (0.504–0.525)	0.615 (0.582–0.647)
ROTEM intem CT	0.506 (0.498–0.514)	0.534 (0.503–0.566)	0.504 (0.497–0.510)	0.539 (0.511–0.568)	0.512 (0.490–0.534)	0.512 (0.446–0.579)	0.506 (0.491–0.520)	0.516 (0.461–0.570)	0.505 (0.496–0.514)	0.541 (0.507–0.575)
ROTEM extem CT	0.517 (0.504–0.530)	0.528 (0.496–0.560)	0.517 (0.506–0.529)	0.534 (0.505–0.563)	0.502 (0.480–0.524)	0.514 (0.451–0.576)	0.533 (0.505–0.561)	0.526 (0.469–0.583)	0.519 (0.504–0.534)	0.530 (0.496–0.565)
ROTEM heptem CT	^a^	0.517 (0.485–0.549)	^a^	0.523 (0.494–0.552)	^a^	0.510 (0.443–0.578)	^a^	0.523 (0.468–0.579)	^a^	0.524 (0.490–0.558)
ROTEM intem α angle	0.567 (0.543–0.591)	0.592 (0.560–0.623)	0.581 (0.559–0.603)	0.613 (0.584–0.642)	0.544 (0.493–0.595)	0.527 (0.455–0.599)	0.598 (0.553–0.644)	0.629 (0.573–0.684)	0.576 (0.550–0.603)	0.608 (0.574–0.641)
ROTEM intem MCF	0.519 (0.506–0.532)	0.597 (0.566–0.628)	0.525 (0.513–0.537)	0.617 (0.589–0.646)	0.507 (0.482–0.532)	0.524 (0.454–0.595)	0.539 (0.510–0.569)	0.643 (0.591–0.695)	0.523 (0.508–0.538)	0.616 (0.583–0.649)
ROTEM fibtem MCF	0.534 (0.513–0.554)	0.593 (0.562–0.624)	0.543 (0.524–0.561)	0.599 (0.571–0.628)	0.501 (0.463–0.539)	0.511 (0.442–0.580)	0.581 (0.539–0.622)	0.634 (0.581–0.688)	0.543 (0.521–0.566)	0.612 (0.579–0.645)

Abbreviations: AUC, area under the receiver operating characteristic curve; bi, test parameter as binary variable; CCB, clinical concern about bleeding; CIB, clinically important bleeding; con, test parameter as continuous variable; ProCoagulants, large volume procoagulant transfusion; RBC_Transfusion, large volume red cell transfusion.

^a^ No established cut‐off threshold is available.

The results of AUROC for individual laboratory reference tests of coagulation, full blood count, aggregometry, organ dysfunction, and biological aging biomarkers for the outcomes in univariate analyses are shown in eTable 5 in Supporting Information. All AUROC values were <0.7, regardless of input as continuous values or as binary variables based on the published positive test thresholds, except for post‐protamine hemoglobin 0.723 (0.670–0.776) and hematocrit 0.723 (0.670–0.776) for large volume red cell transfusion. Sensitivity analyses restricted to participants who developed clinical concern about bleeding, the subset who were actively bleeding, did not alter the overall conclusion.

### 3.4 Best predictive models using all test parameters

**FIGURE 2:**
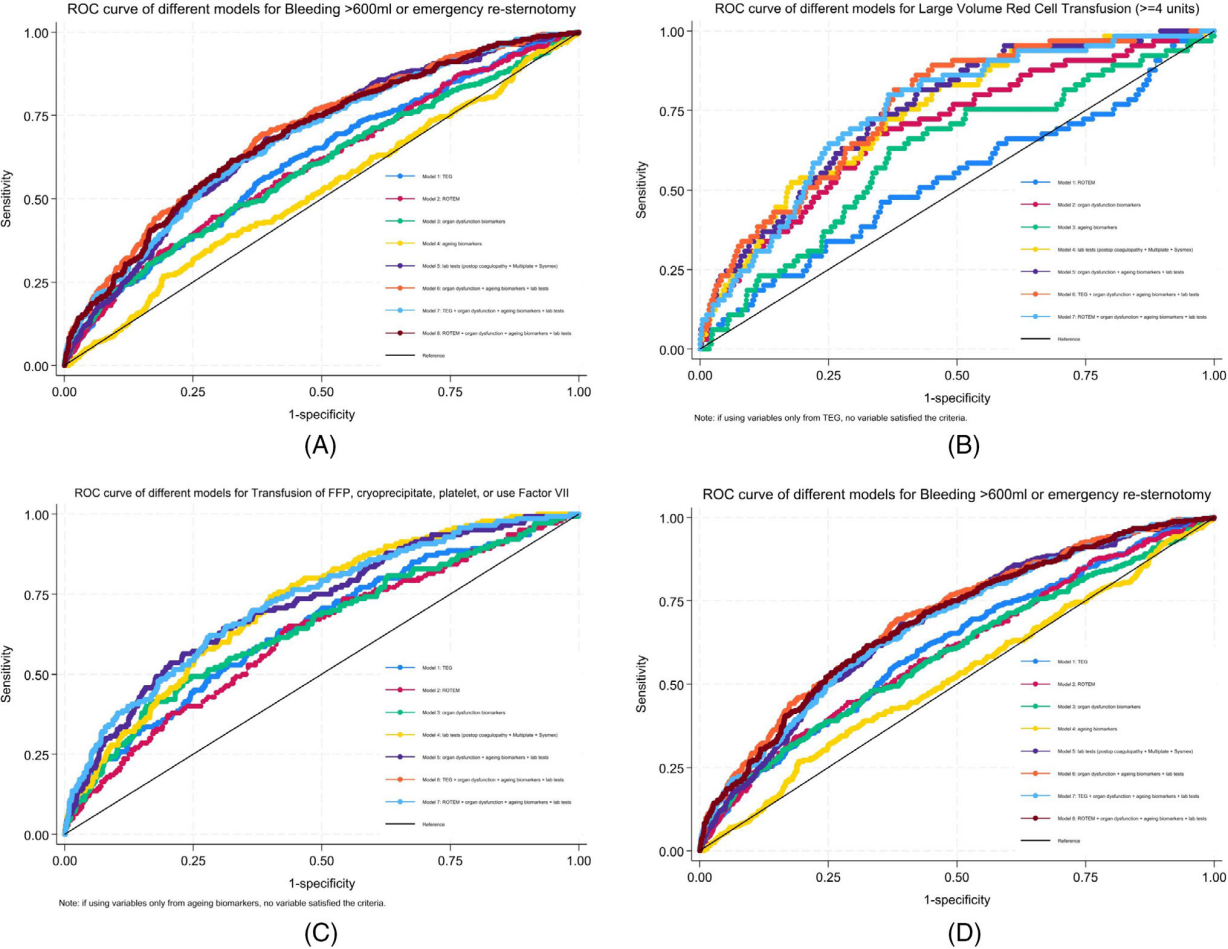
ROC curve showing model discrimination for clinically important bleeding and its three individual components.

**FIGURE 3:**
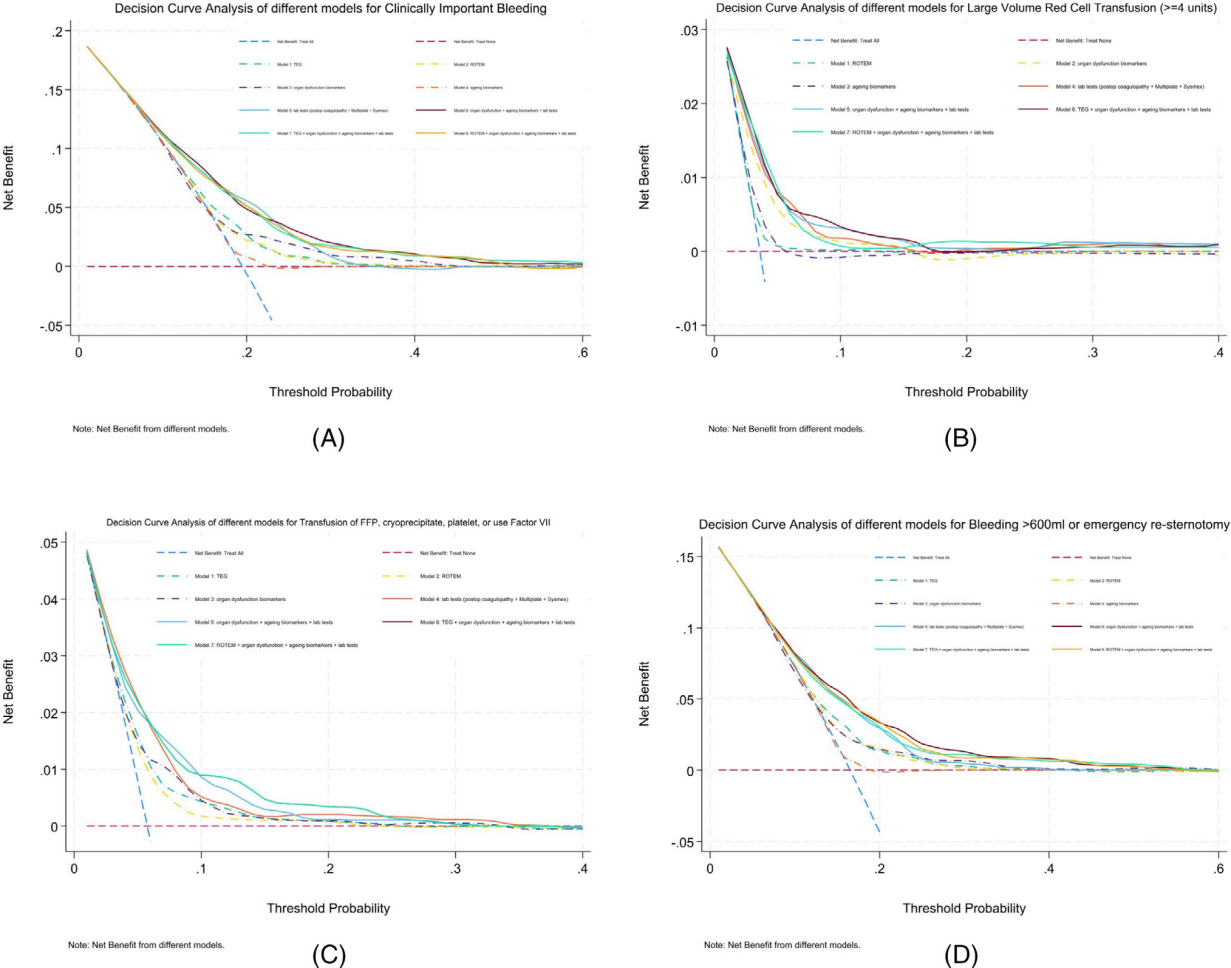
Decision curve analysis for clinically important bleeding and its three individual components.

Multivariable models with different combinations of biomarkers and test parameters to predict the primary outcome and its three components were developed and internally validated using 10‐fold cross‐validation. Predictors in each model for the outcomes are listed in eTable 6 in Supporting Information. ROC curves showing model discrimination and decision curve analysis showing the clinical utility of different models for the primary outcome (A) and its three individual components (B–D) are shown in Figures 2 and 3, respectively. The Brier scores were close among the models for each outcome, and the O:E ratios were close to 1, which indicated good calibration (both in eTable 6 in Supporting Information), meaning the predicted probabilities had high agreement with the observed frequencies.

Models using the predictors from TEG or ROTEM had lower AUROC values (<0.7, Table 3) for the primary outcome and its individual components, as well as worse clinical utility. The model derived from full blood count, aggregometry, and laboratory tests of coagulation demonstrated AUROC 0.688 (0.607–0.769) for the primary outcome, AUROC 0.745 (0.602–0.886) for large volume red cell transfusion, AUROC 0.720 (0.595–0.845) for large volume procoagulant transfusion, and AUROC 0.680 (0.592–0.769) for excessive bleeding.

With additional organ dysfunction biomarkers and aging biomarkers to the laboratory tests, the AUROC values improved slightly for the primary outcome 0.701 (0.620–0.781), large volume red cell transfusion 0.754 (0.602–0.903), large volume procoagulant transfusion 0.723 (0.590–0.857), and excessive bleeding 0.701 (0.613–0.788), but had similar clinical utility. TEG or ROTEM parameters were not selected as predictors in the final multivariable models.

**TABLE 3:** Area under the receiver operating characteristic curve (AUROC, discrimination of prediction models) for clinically important bleeding and its individual components.

Models	Clinically important bleeding	Large volume red cell transfusion	Large volume procoagulant transfusion	Excessive bleeding
TEG	0.611 (0.521–0.701)	^a^	0.649 (0.501–0.798)	0.620 (0.523–0.716)
ROTEM	0.588 (0.496–0.681)	0.580 (0.392–0.766)	0.630 (0.487–0.774)	0.606 (0.508–0.704)
Organ dysfunction biomarkers	0.606 (0.512–0.701)	0.701 (0.501–0.900)	0.651 (0.497–0.805)	0.602 (0.501–0.702)
Aging biomarkers	0.536 (0.433–0.639)	0.646 (0.410–0.880)	^a^	0.530 (0.419–0.641)
Lab tests: post‐op (Sysmex + multiplate + coagulation)	0.688 (0.607–0.769)	0.745 (0.602–0.886)	0.720 (0.595–0.845)	0.680 (0.592–0.769)
Organ dysfunction biomarkers + aging biomarkers + Lab tests	0.701 (0.620–0.781)	0.754 (0.602–0.903)	0.723 (0.590–0.857)	0.701 (0.613–0.788)
TEG + organ dysfunction biomarkers + aging biomarkers + Lab tests	0.694 (0.612–0.775)	0.762 (0.626–0.897)	0.732 (0.606–0.858)	0.686 (0.596–0.776)
ROTEM + Organ dysfunction biomarkers + aging biomarkers + Lab tests	0.692 (0.610–0.775)	0.753 (0.608–0.895)	0.732 (0.606–0.858)	0.695 (0.606–0.783)

*Note*: Values in red color and red highlight, AUROC >0.7.

^a^ No significant predictors for the model.

## 4 DISCUSSION

### 4.1 Main findings

TEG demonstrated moderate to good predictive accuracy, and ROTEM showed good to excellent predictive accuracy for laboratory reference tests of coagulation, low hematocrit, and low platelet counts/volume product, but were poor predictors for clinically important bleeding. FBC, multiplate, and laboratory tests of coagulation also demonstrated poor predictive accuracy for clinically important bleeding. The addition of multimorbidity and biological aging biomarker results did not significantly improve predictive accuracy for clinically important bleeding beyond that observed with the laboratory data.

TEG/ROTEM demonstrated poor predictive accuracy for all three components of the primary outcome. Multiplate, coagulation tests, and FBC data demonstrated moderate predictive accuracy for both large volume red cell transfusion and large volume procoagulant transfusion, but not for excessive bleeding. Additional multimorbidity and biological aging biomarkers yielded small increases in predictive accuracy with similar clinical utility, with the largest AUROC of 0.701 for excessive bleeding among all the prediction models.

### 4.2 Strengths and limitations

First, to our knowledge, COPTIC remains the largest diagnostic test accuracy study of bleeding tests in cardiac surgery.^7^ Second, we used the QUADAS‐2 framework to minimize potential bias. Specifically, the study design avoided the limitations of previous similar studies identified in a systematic review, including non‐prespecified test thresholds, non‐blinded clinical staff, small sample sizes, and limited applicability.^7^ Third, we used regularization techniques (Lasso) to inform the selection of predictors from several dozen biomarkers/test parameters, to get simpler models with fewer parameters for better interpretability and for potential clinical use. We also avoided using “black box” algorithms or yielding overly complex models that may just capture the noise of the data. Finally, we used 10‐fold cross‐validation to internally validate the models and conducted a comprehensive assessment of model performance for discrimination, calibration, and clinical utility.

The first limitation is the age of the study. The COPTIC study recruited participants between 2010 and 2012, and cardiac surgery populations have changed over the last decade. In mitigation, this is the largest available bioresource (*n* = 2437) to our knowledge with simultaneous measurement of multiple disease processes associated with bleeding using high‐quality prospectively collected clinical data and standardized assays performed in a dedicated laboratory. The COPTIC study included many high‐risk procedures, over 90% of all elective and urgent patients operated on in a single large tertiary cardiac center over 2 years, typical of contemporary practice at the time, minimizing applicability bias. We also consider the degradation of stored plasma samples unlikely; baseline biomarker values for multimorbidity and aging in the COPTIC samples were comparable to those from more recent cohorts.^10^ Importantly, this resource enables testing of new hypotheses on the pathogenesis of post‐cardiac surgery bleeding at relatively low cost, although positive findings in any such analysis would require validation in a contemporary study. Second, the study evaluates the TEG 5000 thromboelastograph that has been superseded by the TEG 6s Hemostasis Analyser. In mitigation, the principles underlying both platforms are similar. Essentially, the mechanical attributes of a clot are used to infer specific defects in blood clotting, and the observed differences in predictive accuracy between the two platforms are not clinically significant.^18^, ^19^ The TEG findings were also replicated with the ROTEM device, which demonstrated better predictive accuracy for individual defects in clotting, platelet aggregation, and hematopoiesis than the TEG 5000.

### 4.3 Clinical interpretation

First, the study shows that the measurement of blood‐derived factors involved in clotting could not predict clinically important bleeding well, specifically excessive bleeding, despite using a data‐driven machine learning approach to select the most relevant predictors. One potential explanation, based on Virchow's triad, is that the contribution of the vessel wall may represent an important unmeasured determinant of bleeding. Preliminary evidence to support this comes from a recent study where we showed that a patient cluster with increased bleeding had significant reductions in ICAM‐1 levels in plasma, indicative of altered vascular activation,^10^ something we did not measure in the current study. In other clinical settings, like trauma, in vitro data show that both endothelial dysfunction and coagulation factor deficiencies result in coagulopathic bleeding, but it is the former that drives the coagulopathy and not the latter. Vascular dysfunction also underlies organ injury following cardiopulmonary bypass,^20^, ^21^, ^22^ providing a new hypothesis for the bleeding paradox for future research. Going forward, markers of endothelial damage should be included in the future models of detecting and treating coagulopathic bleeding, in addition to current coagulation tests.

Second, these observations lead us to hypothesize that the three components of the primary outcome represent different mechanisms. This is supported by the Venn diagram and the differences in the biomarkers showing various predictive accuracy for each component. This suggests that composite definitions of clinically important bleeding in this and previous studies,^5^, ^6^ or the use of the individual components interchangeably,^1^, ^2^ are likely barriers to understanding the underlying processes. This information will assist in further attempts to identify mechanisms or phenotypes underlying bleeding and organ dysfunction, and enable the development of targeted treatments.

## 5 CONCLUSION

Current diagnostic tests demonstrate moderate predictive accuracy for excessive bleeding following cardiac surgery. Additional biomarkers of multimorbidity, organ dysfunction, and biological aging did not improve discrimination or clinical utility substantially. Revised definitions of excessive bleeding phenotypes based on these results should reduce barriers to the understanding of underlying disease processes.

## AUTHOR CONTRIBUTIONS

G.J.M. and W.L. designed the study. W.L. performed the statistical analyses. G.J.M. and W.L. interpreted the results and drafted the manuscript. G.J.M. is the senior author and guarantor of the study. A.M. designed the COPTIC study and was the chief investigator of the COPTIC A, B, and COPTIC LRT studies and led the laboratory analyses of the data subsequently used in this study. R.G. and L.G. co‐designed the study and interpreted the study data. M.W. designed and supervised the laboratory analyses of multimorbidity and biological aging biomarkers. H.P. undertook the laboratory analyses. F.L. performed some of the statistical analyses. H.A. was the coordinator of the study. All authors approved the final manuscript for submission.

## FUNDING INFORMATION

G.J.M., W.L., H.A., Y.L., and M.W. are funded by British Heart Foundation CH/12/1/29419. The COPTIC Study was funded by the National Institute for Health and Care Research (NIHR, ref.: RP‐PG‐0407‐10384). R.G. is funded by the NIHR Leicester Biomedical Research Centre (ref: NIHR203327), and the biomarker assays were part funded by this grant, along with the British Heart Foundation Leicester Accelerator Award AA/18/3/34220, and British Heart Foundation Grant RG/17/9/32812. L.G. is funded by NHS Blood and Transplant. The funders have no role in data collection, analysis, interpretation of findings, writing the manuscript, or the decision to submit and publish this article.

## CONFLICT OF INTEREST STATEMENT

G.J.M. declares a financial relationship with Pharmacosmos. Other authors have no conflicts of interest to declare.

## Supporting information


**APPENDIX S1:** Supporting information.

**APPENDIX S2:** Supporting information.

## Data Availability

Research data are not shared.
