# Supplementary material for: Predictive accuracy of diagnostic tests for excessive bleeding in cardiac surgery: The COPTIC‐C study
Source: Transfusion. 2025 Oct 19;65(11):2077–90. doi: 10.1111/trf.18399 (PMC12618898; doi:10.1111/trf.18399)
Supplement: Supplementary file 1 — APPENDIX S1: Supporting information. [file TRF-65-2077-s001.pdf]

Supplementary materials

Predictive accuracy of diagnostic tests for excessive bleeding in cardiac surgery: the COPTIC-C study

[1] Weiqi Liao (Senior Statistician) ORCID: 0000-0002-8605-3749

[1] Robert Grant (Clinical Research Fellow)

[1] Florence Y Lai (Senior Statistician)

[1] Hardeep Aujla (Senior Research Manager)

[1] Marcin Wozniak (Lecturer in Cardiovascular Sciences)

[1] Hasmukh R Patel (Senior Laboratory Technician)

[2,3] Laura Green (Professor of Haemostasis and Transfusion Medicine) ORCID: 0000-0003-4063-9768

[4] Andrew Mumford (Professor of Haematology)

[1] Gavin J Murphy (British Heart Foundation Chair of Cardiac Surgery, Chief Investigator)

Affiliations

[1] Department of Cardiovascular Sciences, University of Leicester, UK

[2] Blizard Institute, Queen Mary University of London, UK

[3] NHS Blood and Transplant, London, UK

[4] Bristol Heart Institute, University of Bristol, UK

Corresponding Author

Dr Weiqi Liao, Senior Statistician, Department of Cardiovascular Sciences, University of Leicester, Clinical Sciences Wing, Glenfield Hospital, Leicester LE3 9QP. Email: weiqi.liao@leicester.ac.uk

Tables for the Methods section

eTable A – The thresholds for Pre-Surgery and Post-Protamine Sysmex testing and Multiplate Aggregometry, and Post-Protamine Laboratory Tests for Coagulopathy

Test	Assay	Units	Interpretation of an abnormal result	Thresholds
**Sysmex tests**				
Haemoglobin (hb)	Sysmex XE-2100	g/dL	Low test result	Male <13.7<br>Female <12.0
Haematocrit (HCt)	Sysmex XE-2100	%	Low test result	Male <0.40<br>Female <0.37
Absolute neutrophils	FBC (XE-2100 cell counter)	$X10^9/L$	Low test result	<1
Lymphocyte count	FBC (XE-2100 cell counter)	$X10^9/L$	Low test result	<0.5
Monocyte count	FBC (XE-2100 cell counter)	$X10^9/L$	High test result	>0.8
Neutrophil/Lymphocyte Ratio	Sysmex XE-2100 (Milton Keynes, UK)		High test result	>3
Mean corpuscular volume	FBC (XE-2100 cell counter)	fl	Low test result	<80
Mean corpuscular haemoglobin	FBC (XE-2100 cell counter)	pg	Low test result	<27
Post-op Absolute reticulocytes	Sysmex XE-2100	%	High test result	<0.68
Post-op PLT	Sysmex XE-2100	$X10^9/L$	Low test result	<140
Post-op MPV	Sysmex XE-2100	fL	Low test result	<9.32
Post-op IPF	Sysmex XE-2100	%	High test result	>4.32
Post-op MPV x Platelets	Sysmex XE-2100	$fL.X10^9/L$	Low test result	<140
**Multiplate tests**				
AUC TRAP-test	Multiplate	Aggregation units (U)	Low test result	<50
AUC ASPI-test	Multiplate	Aggregation units (U)	Low test result	<30
AUC ADP-test	Multiplate	Aggregation units (U)	Low test result	<30

Post Protamine Laboratory Tests of Coagulopathy				
Post-op Prothrombin Time (PT)	Innovin	Seconds (s)	High test result	>15
Post-op Activated Partial Thromboplastin Time (APTT)	actin FS	Seconds (s)	High test result	>35
Claus Fibrinogen (Factor I)	Clauss Fibrinogen activity (thrombin reagent)	g/L	Low test result	<2
anti-Xa	Hyphen Biomed anti-Xa kit	IU/mL	High test result	>1.0
DDIC	Innovance D-dimer assay	ng/mL	High test result	>500
FXIII	FXIII activity (Berichrom XIII),	IU/dL	Low test result	<60
vWF	vWF Risctocetin cofactor activity (BC RiCof assay)	IU/dL	Low test result	<50
ETP (Factor II)	FluCa-kit (Stago, Asnieres sur Seine, France)	nM/min	Low test result	<1336

Abbreviation

- AUC = area under the curve

eTable B – Post-Protamine TEG criteria for a positive test result

Parameters	Units	Interpretation of an abnormal result	Thresholds
TEG CK R	Mm	High test result	> 10
TEG CK α angle	Degrees	Low test result	< 37.6
TEG CK MA	Mm	Low test result	< 44
TEG CKH R	Minutes (mm)	High test result	N/A

Abbreviations

- α-Angle: measures the speed of fibrin build-up
- CK: citrated kaolin
- CKH: citrated kaolin with heparinise
- MA: maximum amplitude of the fibrin clot
- R (min): reaction time (in minutes) to initial fibrin formation

eTable C – Post-Protamine ROTEM criteria for a positive test result

Parameter	Units	Interpretation of an abnormal result	Thresholds
**INTEM**			
intem CT	Minutes (min)	High test result	>240
intem α angle	Degrees	Low test result	<70
intem MCF	Mm	Low test result	<50
**EXTEM**			
extem CT	Minutes (m)	High test result	>79
**FIBTEM**			
fibtem MCF	Mm	Low test result	<9
**HEPTEM**			
heptem CT	Minutes (min)	High test result	N/A

Abbreviations

- CT: clotting time
- MCF: maximum clot firmness

eTable D – Baseline biomarkers of Organ Dysfunction and Biological Ageing

Biomarkers	Units	Interpretation of an abnormal result	Thresholds
**Haematopoiesis**			
Baseline Haemoglobin	g/dL	Low test result	Male <13.7<br>Female <12.0
Baseline haematocrit	%	Low test result	Male <0.40<br>Female< 0.37
Baseline Absolute Reticulocyte	%	High test result	<0.68
Lymphocyte count	$X10^9/L$	Low test result	<0.2
Baseline monocyte count	$X10^9/L$	High test result	>0.8
Baseline neutrophil/lymphocyte ratio		High test result	>3
Transferrin	g/L		
Iron	µmol/L		<10
Transferrin Saturation	%	Low test result	<20%
Serum Ferritin	ng/mL		Adult male <25<br>Adult female <10
Baseline Platelet count	$X10^9/L$	Low test result	<140
Post-op MPV	fL	Low test result	<9.32
Post-op IPF	%	High test result	>4.32
Post-op MPV x Platelets	fL	Low test result	<140
**Liver**			
Bilirubin	µmol/L	High test result	>20
Albumin	g/L	Low test result	<34
**Bone**			
Alkaline Phosphatase	IU/L	High test result	>147
**Kidney**			
eGFR	mls/min×1.73m^2	Low test result	<60
**Inflammation**			
CRP	pg/mL	High test result	>5
Interleukin 6	pg/mL	High test result	>43.5

Interleukin 8	pg/mL	N/A
**Biological Ageing**		
CCL11/Eotaxin	pg/mL	N/A
CX3CL1/Fractalkine	pg/mL	N/A
GDF-15/Mito dysfunction	pg/mL	N/A
CXCL9/iAge	pg/mL	N/A

eTable 1 – Characteristics of the COPTIC participants who were included or excluded from the analysis dataset.

	Patients in the COPTIC study		
	**Included in analysis**	**Consented patients excluded from analysis**	**P value**
N (%)	2,437 (98.5%)	38 (1.5%)	
Age at surgery	66.74 (11.84)	61.19 (14.67)	0.005
Sex			
Male	1,836 (75.3%)	29 (76.3%)	0.890
Female	601 (24.7%)	9 (23.7%)	
Ethnicity			
White	2,301 (94.4%)	35 (97.2%)	0.702
Asian/Black/Mixed	35 (1.4%)	0 (0.0%)	
Unknown	101 (4.1%)	1 (2.8%)	
BMI	27.96 (4.75)	27.34 (5.55)	0.446
BMI>=30			
No	1,672 (70.0%)	26 (74.3%)	0.579
Yes	718 (30.0%)	9 (25.7%)	
Smoking history			
Never smoked	937 (38.6%)	11 (31.4%)	0.630
Ex-smoker	1,399 (57.6%)	22 (62.9%)	
Current smoker	94 (3.9%)	2 (5.7%)	
NYHA>=3			
No	1,654 (68.1%)	27 (77.1%)	0.255
Yes	774 (31.9%)	8 (22.9%)	
Previous MI (Binary)			
No	1,622 (67.0%)	22 (62.9%)	0.603
Yes	798 (33.0%)	13 (37.1%)	
CCS>=2			
No	1,102 (45.4%)	16 (45.7%)	0.967
Yes	1,327 (54.6%)	19 (54.3%)	
Previous PCI			
No	2,207 (91.0%)	33 (94.3%)	0.496
Yes	219 (9.0%)	2 (5.7%)	
Ejection fraction category			
Good (>50%)	1,908 (78.6%)	28 (77.8%)	0.698
Fair (30-49%)	392 (16.1%)	5 (13.9%)	
Poor (<30%)	129 (5.3%)	3 (8.3%)	
Anaemia			
No	1,478 (61.1%)	22 (62.9%)	0.830
Yes	942 (38.9%)	13 (37.1%)	
CKD (baseline eGFR<60)			
No	1,721 (70.8%)	0 (0.0%)	0.119
Yes	709 (29.2%)	1 (100.0%)	
Diabetes			
No	1,939 (79.8%)	32 (91.4%)	0.275
Diet controlled	87 (3.6%)	0 (0.0%)	
Oral therapy controlled	263 (10.8%)	1 (2.9%)	
Insulin controlled	140 (5.8%)	2 (5.7%)	
Hypertension			
No	707 (29.2%)	13 (37.1%)	0.306
Yes	1,714 (70.8%)	22 (62.9%)	
Respiratory disease			
No	2,128 (87.5%)	30 (85.7%)	0.746
Yes	303 (12.5%)	5 (14.3%)	
Cerebrovascular disease			
No	2,204 (91.0%)	33 (94.3%)	0.499
Yes	218 (9.0%)	2 (5.7%)	

History of peripheral vascular disease			
No	2,201 (90.5%)	32 (91.4%)	0.858
Yes	230 (9.5%)	3 (8.6%)	
History of neurological dysfunction			
No	2,367 (97.4%)	33 (94.3%)	0.262
Yes	64 (2.6%)	2 (5.7%)	
Acute kidney injury (AKI)			
No AKI	1,345 (55.6%)	0 (0.0%)	0.004
AKI Stage 1	746 (30.8%)	0 (0.0%)	
AKI Stage 2	162 (6.7%)	0 (0.0%)	
AKI Stage 3	167 (6.9%)	1 (100.0%)	
PhenoAge	66.51 (13.38)	. (.)	.
CABG surgery			
No	463 (31.2%)	8 (38.1%)	0.496
Yes	1,023 (68.8%)	13 (61.9%)	
Valve surgery			
No	869 (58.5%)	14 (66.7%)	0.449
Yes	617 (41.5%)	7 (33.3%)	
Both CABG and Valve			
No	1,289 (86.7%)	20 (95.2%)	0.252
Yes	197 (13.3%)	1 (4.8%)	
Aortic surgery			
No	1,410 (94.9%)	20 (95.2%)	0.942
Yes	76 (5.1%)	1 (4.8%)	
Cumulative bypass time	65.98 (68.02)	58.20 (60.13)	0.501

Note: Mean (Standard deviation) for continuous variables, N (%) for binary and categorical variables.

eTable 2 – Descriptive statistics and uni-variable logistic regression of tests in participants with and without clinically important bleeding

	Clinically Important Bleeding				
	**No**	**Yes**	**Total**	**Odds Ratio (95% CI)**	**P value**
N (%)	1,960 (80.4%)	477 (19.6%)	2,437 (100.0%)		
TEG CK rmin (con)	6.18 (1.95)	6.21 (2.31)	6.18 (2.02)	1.01 (0.96 - 1.06)	0.782
CKH rmin (con)	5.90 (1.37)	5.85 (1.38)	5.89 (1.37)	0.97 (0.90 - 1.05)	0.521
TEG CK α angle (con)	62.59 (7.18)	61.84 (7.76)	62.45 (7.30)	0.99 (0.97 - 1.00)	0.062
TEG CK MA (con)	59.18 (6.43)	56.84 (6.92)	58.74 (6.59)	0.95 (0.93 - 0.96)	<0.001
ROTEM intem CT (con)	169.14 (34.85)	173.14 (32.07)	169.90 (34.37)	1.00 (1.00 - 1.01)	0.048
ROTEM extem CT (con)	57.49 (11.82)	59.23 (13.75)	57.82 (12.22)	1.01 (1.00 - 1.02)	0.010
ROTEM heptem CT (con)	168.63 (28.60)	171.13 (31.75)	169.10 (29.23)	1.00 (1.00 - 1.01)	0.123
ROTEM intem α angle (con)	73.77 (5.00)	71.76 (6.34)	73.39 (5.34)	0.94 (0.92 - 0.96)	<0.001
ROTEM intem MCF (con)	61.60 (6.15)	59.31 (6.77)	61.17 (6.34)	0.94 (0.93 - 0.96)	<0.001
ROTEM fibtem MCF (con)	14.22 (5.38)	12.77 (5.47)	13.95 (5.42)	0.95 (0.92 - 0.97)	<0.001
post-op HB (con)	10.06 (1.64)	9.33 (1.46)	9.92 (1.63)	0.74 (0.69 - 0.80)	<0.001
post-op Haematocrit (con)	0.29 (0.05)	0.27 (0.04)	0.28 (0.05)	0.00 (0.00 - 0.00)	<0.001
post-op lymphocyte count (con)	1.47 (1.85)	1.25 (1.50)	1.42 (1.79)	0.74 (0.63 - 0.88)	0.001
post-op monocyte count (con)	0.34 (0.28)	0.34 (0.94)	0.34 (0.48)	0.96 (0.73 - 1.25)	0.753
post-op neutrophil/lymphocyte Ratio (con)	6.48 (4.60)	7.62 (5.58)	6.70 (4.82)	1.04 (1.02 - 1.07)	<0.001
post-op mean corpuscular volume (con)	85.59 (5.01)	85.95 (5.22)	85.66 (5.05)	1.01 (0.99 - 1.04)	0.183
post-op mean corpuscular haemoglobin (con)	30.05 (1.82)	30.28 (1.89)	30.09 (1.83)	1.07 (1.01 - 1.14)	0.020
post-op Absolute reticulocytes (con)	41.84 (17.02)	39.19 (17.50)	41.33 (17.14)	0.99 (0.98 - 1.00)	0.004
post-op Platelets (con)	149.21 (51.22)	133.01 (45.77)	146.13 (50.62)	0.99 (0.99 - 1.00)	<0.001
post-op MPV (con)	10.44 (0.99)	10.29 (0.97)	10.41 (0.99)	0.85 (0.76 - 0.95)	0.004
post-op IPF (con)	3.58 (2.29)	3.56 (2.26)	3.58 (2.28)	0.99 (0.95 - 1.04)	0.824
Post-op MPV X Platelets (con)	1,547.51 (511.75)	1,361.36 (457.91)	1,512.04 (507.14)	1.00 (1.00 - 1.00)	<0.001
Post-op PT (con)	12.43 (1.11)	12.79 (1.32)	12.50 (1.16)	1.28 (1.17 - 1.39)	<0.001
Post-op APTT (con)	32.22 (6.80)	34.75 (9.88)	32.72 (7.58)	1.04 (1.02 - 1.06)	<0.001
Anti-Xa heparin (con)	0.19 (0.14)	0.20 (0.16)	0.20 (0.14)	1.31 (0.64 - 2.68)	0.467
Endogenous thrombin potential (con)	1,044.43 (479.82)	991.55 (460.06)	1,033.87 (476.30)	1.00 (1.00 - 1.00)	0.042
Clauss Fibrinogen (con)	2.24 (0.77)	1.93 (0.72)	2.18 (0.77)	0.53 (0.45 - 0.63)	<0.001
D dimer (con)	691.16 (1,023.28)	881.20 (1,277.26)	728.91 (1,080.84)	1.00 (1.00 - 1.00)	0.002
Factor XIII (con)	81.58 (20.30)	74.78 (19.08)	80.23 (20.24)	0.98 (0.98 - 0.99)	<0.001
Von Willebrand factor ristocetin cofactor (con)	159.02 (59.32)	161.31 (68.94)	159.48 (61.35)	1.00 (1.00 - 1.00)	0.492
post-op TRAP test AUC (con)	117.66 (39.25)	107.12 (38.86)	115.65 (39.38)	0.99 (0.99 - 1.00)	<0.001

	Clinically Important Bleeding				
	**No**	**Yes**	**Total**	**Odds Ratio (95% CI)**	**P value**
post-op ASPI test AUC (con)	29.32 (25.21)	23.91 (20.93)	28.29 (24.54)	0.99 (0.98 - 0.99)	<0.001
post-op ADP test AUC (con)	57.92 (29.50)	46.40 (25.24)	55.73 (29.09)	0.98 (0.98 - 0.99)	<0.001
Haemoglobin (pre-operative)	13.55 (1.61)	13.27 (1.87)	13.49 (1.66)	0.90 (0.85 - 0.96)	0.001
pre-op Haematocrit (con)	0.36 (0.04)	0.35 (0.05)	0.36 (0.04)	0.00 (0.00 - 0.02)	<0.001
pre-op mean corpuscular volume (con)	85.62 (5.15)	85.79 (5.48)	85.66 (5.22)	1.01 (0.99 - 1.03)	0.535
pre-op mean corpuscular haemoglobin (con)	30.02 (1.90)	30.20 (2.03)	30.06 (1.93)	1.05 (1.00 - 1.11)	0.075
pre-op Absolute reticulocytes (con)	48.43 (19.61)	47.19 (23.28)	48.19 (20.37)	1.00 (0.99 - 1.00)	0.238
Transferrin (con)	1.97 (0.33)	1.93 (0.37)	1.96 (0.34)	0.72 (0.52 - 0.99)	0.046
Transferrin Saturation (con)	25.94 (11.81)	25.91 (11.81)	25.93 (11.81)	1.00 (0.99 - 1.01)	0.968
Iron (con)	12.60 (5.16)	12.28 (5.07)	12.54 (5.14)	0.99 (0.97 - 1.01)	0.272
Ferritin (con)	120.24 (112.13)	138.13 (130.05)	123.72 (116.02)	1.00 (1.00 - 1.00)	0.006
pre-op lymphocyte count (con)	1.84 (2.02)	1.63 (1.20)	1.80 (1.89)	0.75 (0.64 - 0.89)	0.001
pre-op monocyte count (con)	0.61 (0.23)	0.60 (0.28)	0.61 (0.24)	0.81 (0.51 - 1.26)	0.344
pre-op neutrophil/lymphocyte Ratio (con)	2.80 (1.97)	3.20 (2.13)	2.87 (2.01)	1.09 (1.04 - 1.14)	<0.001
pre-op Platelets (con)	212.14 (62.38)	203.27 (64.47)	210.41 (62.88)	1.00 (1.00 - 1.00)	0.006
pre-op IPF (con)	3.22 (2.07)	3.25 (2.27)	3.23 (2.11)	1.01 (0.96 - 1.06)	0.740
pre-op MPV (con)	10.65 (0.99)	10.52 (0.96)	10.63 (0.99)	0.87 (0.78 - 0.97)	0.009
Bilirubin (con)	8.59 (5.52)	9.32 (7.06)	8.73 (5.86)	1.02 (1.00 - 1.04)	0.027
Albumin (con)	36.41 (3.40)	35.68 (3.97)	36.27 (3.53)	0.94 (0.91 - 0.97)	<0.001
Baseline eGFR (con)	71.00 (20.35)	69.09 (20.85)	70.63 (20.46)	1.00 (0.99 - 1.00)	0.067
Alkaline Phosphatase (con)	58.81 (22.99)	63.95 (70.16)	59.81 (37.23)	1.00 (1.00 - 1.01)	0.046
Troponin (con)	9.77 (28.54)	17.75 (42.57)	11.97 (33.11)	1.01 (1.00 - 1.01)	0.110
Interleukin 6 (con)	3.04 (6.91)	2.91 (5.12)	3.02 (6.59)	1.00 (0.98 - 1.01)	0.723
Interleukin 8 (con)	3.73 (8.99)	4.01 (7.43)	3.79 (8.70)	1.00 (0.99 - 1.02)	0.567
CRP (con)	1,621,968.31 (5,029,349.96)	1,940,144.47 (5,168,944.60)	1,684,900.58 (5,057,479.49)	1.00 (1.00 - 1.00)	0.291
CXCL11 (Eotaxin)	152.89 (422.82)	138.22 (220.56)	150.03 (391.68)	1.00 (1.00 - 1.00)	0.511
CX3CL1 (Fractalkine)	70,680.92 (980,080.82)	48,940.11 (553,009.67)	66,448.68 (912,665.22)	1.00 (1.00 - 1.00)	0.680
GDF-15 (mito dysfunction)	859.12 (1,110.19)	977.34 (1,024.68)	882.17 (1,094.80)	1.00 (1.00 - 1.00)	0.069
CXCL9 (iAge)	1,376.95 (4,058.49)	1,462.13 (2,691.89)	1,393.54 (3,830.30)	1.00 (1.00 - 1.00)	0.695

Note: Mean (Standard deviation) for continuous variables.

eTable 3 – Predictive accuracy of TEG for the corresponding Post-Protamine laboratory tests of coagulation/multiplate aggregometry/Sysmex parameters

TEG parameters (index test)	Test parameters	AUROC (Index test as binary variable)	AUROC (Index test as continuous variable)
TEG CK rmin	post-op HB	0.503 (0.476 - 0.529)	0.718 (0.641 - 0.795)
TEG CK rmin	post-op Haematocrit	0.511 (0.508 - 0.514)	0.664 (0.564 - 0.764)
TEG CK rmin	Post-op APTT	0.519 (0.509 - 0.529)	0.619 (0.590 - 0.648)
TEG CK rmin	Post-op PT (14s)	0.508 (0.493 - 0.523)	0.519 (0.471 - 0.568)
TEG CK rmin	Post-op PT (15s)	0.504 (0.489 - 0.519)	0.509 (0.441 - 0.576)
TEG CK rmin	Endogenous thrombin potential	0.515 (0.511 - 0.520)	0.597 (0.570 - 0.624)
CKH rmin	Anti-Xa heparin	*	**0.852 (0.672 - 1.000)**
CKH rmin	Post-op APTT	*	0.607 (0.578 - 0.636)
TEG CK α angle	Endogenous thrombin potential	0.502 (0.499 - 0.505)	0.570 (0.542 - 0.599)
TEG CK α angle	Clauss Fibrinogen	0.503 (0.500 - 0.507)	0.635 (0.611 - 0.659)
TEG CK α angle	post-op TRAP test AUC	0.503 (0.501 - 0.505)	0.563 (0.505 - 0.621)
TEG CK α angle	post-op ASPI test AUC	0.504 (0.502 - 0.507)	0.596 (0.570 - 0.623)
TEG CK α angle	post-op ADP test AUC	0.502 (0.497 - 0.507)	0.576 (0.546 - 0.607)
TEG CK α angle	post-op Platelets	0.503 (0.500 - 0.507)	0.650 (0.627 - 0.673)
TEG CK α angle	post-op MPV	0.506 (0.499 - 0.514)	0.522 (0.486 - 0.559)
TEG CK α angle	post-op IPF	0.504 (0.502 - 0.506)	0.509 (0.482 - 0.537)
TEG CK α angle	Post-op MPV X Platelets	0.501 (0.498 - 0.504)	0.653 (0.629 - 0.676)
TEG CK α angle	Von Willebrand factor ristocetin cofactor	0.503 (0.502 - 0.505)	0.673 (0.523 - 0.822)
TEG CK MA	Clauss Fibrinogen	0.511 (0.506 - 0.517)	**0.829 (0.812 - 0.847)**
TEG CK MA	D dimer	0.502 (0.497 - 0.507)	0.624 (0.599 - 0.649)
TEG CK MA	Factor XIII	0.538 (0.522 - 0.554)	0.741 (0.709 - 0.772)
TEG CK MA	Von Willebrand factor ristocetin cofactor	0.507 (0.504 - 0.509)	0.711 (0.594 - 0.829)
TEG CK MA	post-op Platelets	0.511 (0.506 - 0.515)	0.795 (0.776 - 0.813)
TEG CK MA	post-op MPV	0.502 (0.494 - 0.510)	0.546 (0.510 - 0.582)
TEG CK MA	post-op IPF	0.502 (0.497 - 0.507)	0.515 (0.488 - 0.543)
TEG CK MA	Post-op MPV X Platelets	0.514 (0.508 - 0.520)	**0.808 (0.789 - 0.827)**
TEG CK MA	post-op TRAP test AUC	0.510 (0.492 - 0.528)	0.651 (0.597 - 0.705)
TEG CK MA	post-op ASPI test AUC	0.510 (0.506 - 0.513)	0.633 (0.607 - 0.658)
TEG CK MA	post-op ADP test AUC	0.516 (0.506 - 0.525)	0.680 (0.651 - 0.708)

Note: * No established cut-off threshold is available.

Note: AUROC > 0.7 | **AUROC > 0.8**

eTable 4 – Predictive accuracy of ROTEM for the corresponding Post-Protamine laboratory tests of coagulation/multiplate aggregometry/Sysmex parameters.

ROTEM parameters (index test)	Test parameters	AUROC (Index test as binary variable)	AUROC (Index test as continuous variable)
ROTEM intem CT	post-op HB	0.508 (0.505 - 0.511)	0.564 (0.475 - 0.652)
ROTEM intem CT	post-op Haematocrit	0.508 (0.505 - 0.510)	0.540 (0.426 - 0.654)
ROTEM intem CT	Post-op APTT	0.514 (0.506 - 0.522)	0.679 (0.652 - 0.706)
ROTEM intem CT	Endogenous thrombin potential	0.507 (0.502 - 0.512)	0.535 (0.506 - 0.563)
ROTEM extem CT	post-op HB	0.506 (0.469 - 0.543)	0.555 (0.468 - 0.642)
ROTEM extem CT	post-op Haematocrit	0.523 (0.464 - 0.582)	0.617 (0.526 - 0.707)
ROTEM extem CT	Post-op PT (14s)	0.558 (0.530 - 0.585)	0.743 (0.702 - 0.783)
ROTEM extem CT	Post-op PT (15s)	0.563 (0.518 - 0.608)	0.788 (0.731 - 0.845)
ROTEM extem CT	Endogenous thrombin potential	0.509 (0.500 - 0.518)	0.521 (0.494 - 0.549)
ROTEM heptem CT	Anti-Xa heparin	*	0.765 (0.609 - 0.921)
ROTEM heptem CT	Post-op APTT	*	0.675 (0.648 - 0.702)
ROTEM intem α angle	Endogenous thrombin potential	0.515 (0.496 - 0.535)	0.535 (0.506 - 0.564)
ROTEM intem α angle	Clauss Fibrinogen	0.666 (0.649 - 0.682)	**0.848 (0.831 - 0.864)**
ROTEM intem α angle	post-op Platelets	0.667 (0.651 - 0.682)	**0.848 (0.832 - 0.864)**
ROTEM intem α angle	post-op MPV	0.505 (0.481 - 0.529)	0.515 (0.479 - 0.552)
ROTEM intem α angle	post-op IPF	0.526 (0.506 - 0.546)	0.562 (0.535 - 0.589)
ROTEM intem α angle	Post-op MPV X Platelets	0.703 (0.684 - 0.721)	**0.838 (0.821 - 0.855)**
ROTEM intem α angle	post-op TRAP test AUC	0.599 (0.548 - 0.650)	0.638 (0.580 - 0.697)
ROTEM intem α angle	post-op ASPI test AUC	0.563 (0.547 - 0.579)	0.613 (0.588 - 0.639)
ROTEM intem α angle	post-op ADP test AUC	0.607 (0.582 - 0.632)	0.657 (0.627 - 0.687)
ROTEM fibtem MCF	Clauss Fibrinogen	0.617 (0.603 - 0.631)	0.908 (0.895 - 0.920)
ROTEM intem MCF	Clauss Fibrinogen	0.538 (0.530 - 0.547)	**0.871 (0.856 - 0.886)**
ROTEM intem MCF	D dimer	0.502 (0.493 - 0.510)	0.618 (0.592 - 0.643)
ROTEM intem MCF	Factor XIII	0.585 (0.562 - 0.608)	0.758 (0.727 - 0.788)
ROTEM intem MCF	Von Willebrand factor ristocetin cofactor	0.519 (0.515 - 0.523)	0.625 (0.491 - 0.760)
ROTEM intem MCF	post-op Platelets	0.536 (0.528 - 0.544)	**0.848 (0.832 - 0.864)**
ROTEM intem MCF	post-op MPV	0.503 (0.491 - 0.515)	0.529 (0.492 - 0.565)
ROTEM intem MCF	post-op IPF	0.504 (0.495 - 0.514)	0.512 (0.484 - 0.539)
ROTEM intem MCF	Post-op MPV X Platelets	0.546 (0.536 - 0.557)	**0.859 (0.843 - 0.875)**
ROTEM intem MCF	post-op TRAP test AUC	0.537 (0.505 - 0.570)	0.672 (0.617 - 0.727)

ROTEM parameters (index test)	Test parameters	AUROC (Index test as binary variable)	AUROC (Index test as continuous variable)
ROTEM intem MCF	post-op ASPI test AUC	0.522 (0.515 - 0.528)	0.632 (0.606 - 0.657)
ROTEM intem MCF	post-op ADP test AUC	0.544 (0.529 - 0.560)	0.675 (0.646 - 0.704)

Note: * No established cut-off threshold is available.

Note: AUROC > 0.7	**AUROC > 0.8**	AUROC > 0.9

eTable 5 – Diagnostic accuracy of individual tests (continuous and binary) for clinically important bleeding, its three individual components, and the secondary outcome.

Test variable	CIB_bi_AUC	CIB_con_AUC	CCB_bi_AUC	CCB_con_AUC	RBC_Transfusion_bi_AUC
post-op HB	0.510 (0.506 - 0.514)	0.627 (0.599 - 0.655)	0.511 (0.507 - 0.515)	0.643 (0.617 - 0.670)	0.509 (0.507 - 0.512)
post-op Haematocrit	0.506 (0.502 - 0.509)	0.636 (0.607 - 0.664)	0.506 (0.503 - 0.510)	0.646 (0.620 - 0.673)	0.506 (0.504 - 0.508)
post-op mean corpuscular volume	0.506 (0.489 - 0.523)	0.518 (0.486 - 0.549)	0.505 (0.490 - 0.520)	0.540 (0.511 - 0.568)	0.518 (0.478 - 0.557)
post-op mean corpuscular haemoglobin	0.505 (0.493 - 0.517)	0.527 (0.496 - 0.559)	0.503 (0.493 - 0.513)	0.537 (0.509 - 0.565)	0.514 (0.485 - 0.543)
post-op Absolute reticulocytes	0.502 (0.496 - 0.508)	0.556 (0.526 - 0.586)	0.504 (0.498 - 0.510)	0.557 (0.530 - 0.585)	0.501 (0.488 - 0.513)
post-op lymphocyte count	0.518 (0.504 - 0.532)	0.594 (0.565 - 0.624)	0.516 (0.504 - 0.528)	0.605 (0.578 - 0.632)	0.572 (0.528 - 0.615)
post-op monocyte count	0.515 (0.506 - 0.525)	0.553 (0.523 - 0.584)	0.511 (0.501 - 0.520)	0.574 (0.546 - 0.602)	0.501 (0.476 - 0.525)
post-op neutrophil/lymphocyte Ratio	0.528 (0.509 - 0.547)	0.574 (0.544 - 0.605)	0.535 (0.518 - 0.552)	0.577 (0.549 - 0.604)	0.563 (0.535 - 0.590)
post-op Platelets	0.571 (0.545 - 0.597)	0.597 (0.567 - 0.628)	0.591 (0.567 - 0.614)	0.632 (0.604 - 0.659)	0.561 (0.506 - 0.615)
post-op MPV	0.514 (0.495 - 0.533)	0.546 (0.516 - 0.577)	0.520 (0.503 - 0.538)	0.529 (0.501 - 0.558)	0.525 (0.482 - 0.568)
post-op IPF	0.515 (0.492 - 0.538)	0.508 (0.477 - 0.538)	0.516 (0.494 - 0.538)	0.523 (0.495 - 0.551)	0.504 (0.454 - 0.554)
Post-op MPV X Platelets	0.585 (0.559 - 0.612)	0.611 (0.581 - 0.642)	0.604 (0.580 - 0.628)	0.640 (0.612 - 0.667)	0.575 (0.519 - 0.630)
Post-op PT	Two cut-offs used below	0.592 (0.562 - 0.623)	Two cut-offs used below	0.593 (0.564 - 0.622)	Two cut-offs used below
Post-op PT (14s)	0.539 (0.522 - 0.557)		0.533 (0.518 - 0.549)		0.551 (0.508 - 0.595)
Post-op PT (15s)	0.514 (0.503 - 0.526)		0.512 (0.501 - 0.522)		0.529 (0.497 - 0.561)
Post-op APTT	0.561 (0.536 - 0.586)	0.601 (0.570 - 0.631)	0.555 (0.532 - 0.578)	0.592 (0.564 - 0.621)	0.536 (0.482 - 0.589)
Anti-Xa heparin	0.503 (0.499 - 0.507)	0.502 (0.470 - 0.534)	0.503 (0.499 - 0.506)	0.511 (0.482 - 0.540)	0.501 (0.500 - 0.502)
Endogenous thrombin potential	0.526 (0.503 - 0.549)	0.529 (0.499 - 0.560)	0.500 (0.478 - 0.523)	0.505 (0.476 - 0.534)	0.534 (0.487 - 0.581)
Clauss Fibrinogen	0.587 (0.561 - 0.613)	0.628 (0.598 - 0.659)	0.590 (0.566 - 0.615)	0.631 (0.603 - 0.660)	0.539 (0.482 - 0.596)
D dimer	0.538 (0.511 - 0.564)	0.544 (0.512 - 0.576)	0.532 (0.507 - 0.557)	0.541 (0.511 - 0.571)	0.594 (0.538 - 0.650)
Factor XIII	0.542 (0.521 - 0.562)	0.606 (0.577 - 0.636)	0.552 (0.532 - 0.571)	0.626 (0.598 - 0.654)	0.515 (0.473 - 0.557)
Von Willebrand factor ristocetin cofactor	0.500 (0.497 - 0.504)	0.500 (0.468 - 0.532)	0.500 (0.497 - 0.504)	0.511 (0.481 - 0.540)	0.502 (0.501 - 0.504)
post-op TRAP test AUC	0.523 (0.510 - 0.537)	0.571 (0.541 - 0.602)	0.514 (0.503 - 0.525)	0.587 (0.559 - 0.615)	0.537 (0.501 - 0.573)
post-op ASPI test AUC	0.547 (0.524 - 0.570)	0.570 (0.540 - 0.600)	0.551 (0.530 - 0.571)	0.580 (0.553 - 0.608)	0.533 (0.485 - 0.582)
post-op ADP test AUC	0.577 (0.553 - 0.602)	0.620 (0.590 - 0.650)	0.591 (0.569 - 0.612)	0.642 (0.615 - 0.669)	0.567 (0.514 - 0.619)
Haemoglobin (pre-operative)	0.556 (0.531 - 0.581)	0.548 (0.519 - 0.577)	0.539 (0.515 - 0.563)	0.531 (0.503 - 0.560)	0.624 (0.570 - 0.678)
pre-op Haematocrit	0.543 (0.523 - 0.563)	0.581 (0.551 - 0.611)	0.521 (0.501 - 0.542)	0.543 (0.514 - 0.572)	0.583 (0.551 - 0.616)
pre-op mean corpuscular volume	0.504 (0.488 - 0.521)	0.508 (0.478 - 0.538)	0.502 (0.486 - 0.518)	0.531 (0.502 - 0.560)	0.516 (0.476 - 0.556)
pre-op mean corpuscular haemoglobin	0.501 (0.490 - 0.513)	0.523 (0.493 - 0.553)	0.502 (0.491 - 0.512)	0.539 (0.510 - 0.568)	0.512 (0.483 - 0.542)
pre-op Absolute reticulocytes	0.501 (0.494 - 0.508)	0.536 (0.507 - 0.566)	0.501 (0.494 - 0.507)	0.522 (0.494 - 0.550)	0.510 (0.489 - 0.532)

Test variable	CIB_bi_AUC	CIB_con_AUC	CCB_bi_AUC	CCB_con_AUC	RBC_Transfusion_bi_AUC
Transferrin	0.500 (0.500 - 0.501)	0.536 (0.503 - 0.568)	0.500 (0.500 - 0.501)	0.510 (0.480 - 0.541)	0.500 (0.500 - 0.501)
Transferrin Saturation	0.502 (0.477 - 0.527)	0.506 (0.474 - 0.537)	0.511 (0.487 - 0.535)	0.504 (0.474 - 0.534)	0.574 (0.514 - 0.634)
Iron	0.505 (0.479 - 0.531)	0.520 (0.489 - 0.551)	0.509 (0.485 - 0.534)	0.500 (0.470 - 0.531)	0.584 (0.523 - 0.644)
Ferritin	0.508 (0.494 - 0.522)	0.542 (0.511 - 0.573)	0.505 (0.491 - 0.520)	0.535 (0.504 - 0.566)	0.513 (0.484 - 0.542)
pre-op lymphocyte count	0.505 (0.499 - 0.510)	0.578 (0.549 - 0.607)	0.504 (0.499 - 0.508)	0.569 (0.541 - 0.597)	0.503 (0.501 - 0.504)
pre-op monocyte count	0.507 (0.489 - 0.524)	0.528 (0.498 - 0.559)	0.502 (0.485 - 0.519)	0.518 (0.490 - 0.547)	0.518 (0.476 - 0.561)
pre-op neutrophil/lymphocyte Ratio	0.543 (0.518 - 0.567)	0.556 (0.526 - 0.586)	0.532 (0.508 - 0.555)	0.541 (0.512 - 0.569)	0.555 (0.500 - 0.611)
pre-op Platelets	0.528 (0.511 - 0.544)	0.545 (0.515 - 0.576)	0.524 (0.509 - 0.540)	0.564 (0.536 - 0.592)	0.506 (0.473 - 0.540)
pre-op IPF	0.503 (0.483 - 0.523)	0.502 (0.472 - 0.531)	0.506 (0.486 - 0.526)	0.517 (0.489 - 0.545)	0.520 (0.478 - 0.562)
pre-op MPV	0.507 (0.493 - 0.522)	0.538 (0.510 - 0.567)	0.511 (0.497 - 0.525)	0.517 (0.489 - 0.546)	0.524 (0.486 - 0.562)
Bilirubin	0.516 (0.503 - 0.528)	0.520 (0.489 - 0.552)	0.510 (0.499 - 0.521)	0.525 (0.495 - 0.555)	0.527 (0.492 - 0.561)
Albumin	0.536 (0.512 - 0.560)	0.542 (0.510 - 0.574)	0.518 (0.495 - 0.540)	0.517 (0.486 - 0.547)	0.568 (0.510 - 0.626)
Baseline eGFR	*	0.524 (0.495 - 0.553)	*	0.532 (0.504 - 0.560)	*
Alkaline Phosphatase	0.506 (0.498 - 0.513)	0.501 (0.469 - 0.534)	0.507 (0.500 - 0.514)	0.503 (0.472 - 0.534)	0.508 (0.488 - 0.529)
Troponin	0.523 (0.484 - 0.563)	0.558 (0.476 - 0.641)	0.520 (0.478 - 0.562)	0.582 (0.500 - 0.664)	0.530 (0.440 - 0.621)
Interleukin 6	0.502 (0.498 - 0.505)	0.508 (0.475 - 0.541)	0.501 (0.499 - 0.504)	0.503 (0.472 - 0.534)	0.502 (0.501 - 0.504)
Interleukin 8	*	0.529 (0.496 - 0.561)	*	0.505 (0.474 - 0.536)	*
CRP	*	0.519 (0.486 - 0.553)	*	0.517 (0.485 - 0.549)	*
CXCL11 (Eotaxin)	*	0.521 (0.489 - 0.553)	*	0.501 (0.470 - 0.532)	*
CX3CL1 (Fractalkine)	*	0.543 (0.511 - 0.575)	*	0.518 (0.487 - 0.549)	*
GDF-15 (mito dysfunction)	*	0.551 (0.518 - 0.583)	*	0.541 (0.510 - 0.572)	*
CXCL9 (iAge)	*	0.538 (0.505 - 0.570)	*	0.519 (0.489 - 0.550)	*
CXCL9 log transformation	*	0.538 (0.505 - 0.570)	*	0.519 (0.489 - 0.550)	*

Note: * No established cut-off threshold is available.
CIB – Clinically important bleeding
CCB – Clinical Concern about Bleeding
RBC_Transfusion – Large volume red cell transfusion
ProCoagulants – Large volume procoagulant transfusion
AUC – area under the receiver operating characteristic curve

bi – test parameter as binary variable
con – test parameter as continuous variable

Note: AUROC > 0.7

eTable 5 – Diagnostic accuracy of individua

Test variable	RBC_Transfusion_con_AUC	ProCoagulants_bi_AUC	ProCoagulants_con_AUC	Excessive bleeding_bi_AUC
post-op HB	0.723 (0.670 - 0.776)	0.510 (0.507 - 0.512)	0.591 (0.546 - 0.637)	0.509 (0.505 - 0.513)
post-op Haematocrit	0.723 (0.670 - 0.776)	0.506 (0.504 - 0.508)	0.614 (0.568 - 0.659)	0.505 (0.501 - 0.509)
post-op mean corpuscular volume	0.518 (0.451 - 0.586)	0.526 (0.491 - 0.560)	0.552 (0.494 - 0.610)	0.503 (0.485 - 0.521)
post-op mean corpuscular haemoglobin	0.541 (0.477 - 0.605)	0.502 (0.482 - 0.523)	0.503 (0.445 - 0.561)	0.504 (0.491 - 0.517)
post-op Absolute reticulocytes	0.545 (0.478 - 0.611)	0.503 (0.491 - 0.515)	0.541 (0.485 - 0.596)	0.500 (0.494 - 0.506)
post-op lymphocyte count	0.641 (0.575 - 0.706)	0.519 (0.493 - 0.545)	0.597 (0.543 - 0.651)	0.510 (0.496 - 0.523)
post-op monocyte count	0.501 (0.433 - 0.569)	0.513 (0.498 - 0.529)	0.553 (0.501 - 0.605)	0.512 (0.501 - 0.522)
post-op neutrophil/lymphocyte Ratio	0.697 (0.637 - 0.757)	0.542 (0.513 - 0.571)	0.587 (0.535 - 0.639)	0.521 (0.500 - 0.542)
post-op Platelets	0.594 (0.526 - 0.662)	0.619 (0.577 - 0.661)	0.655 (0.604 - 0.706)	0.578 (0.550 - 0.606)
post-op MPV	0.577 (0.514 - 0.640)	0.527 (0.490 - 0.563)	0.546 (0.491 - 0.601)	0.510 (0.489 - 0.530)
post-op IPF	0.504 (0.440 - 0.568)	0.505 (0.463 - 0.547)	0.504 (0.449 - 0.560)	0.509 (0.484 - 0.534)
Post-op MPV X Platelets	0.616 (0.549 - 0.683)	0.627 (0.582 - 0.673)	0.664 (0.613 - 0.715)	0.594 (0.566 - 0.622)
Post-op PT	0.600 (0.536 - 0.665)	Two cut-offs used below	0.597 (0.544 - 0.651)	Two cut-offs used below
Post-op PT (14s)		0.560 (0.525 - 0.595)		0.534 (0.515 - 0.553)
Post-op PT (15s)		0.508 (0.489 - 0.527)		0.510 (0.498 - 0.522)
Post-op APTT	0.576 (0.514 - 0.639)	0.565 (0.521 - 0.609)	0.626 (0.576 - 0.676)	0.566 (0.539 - 0.593)
Anti-Xa heparin	0.529 (0.461 - 0.598)	0.503 (0.495 - 0.511)	0.536 (0.479 - 0.592)	0.504 (0.499 - 0.509)
Endogenous thrombin potential	0.505 (0.443 - 0.567)	0.527 (0.489 - 0.565)	0.525 (0.475 - 0.575)	0.517 (0.492 - 0.542)
Clauss Fibrinogen	0.551 (0.482 - 0.619)	0.622 (0.580 - 0.663)	0.681 (0.633 - 0.730)	0.599 (0.571 - 0.626)
D dimer	0.622 (0.555 - 0.689)	0.501 (0.456 - 0.547)	0.523 (0.470 - 0.577)	0.534 (0.505 - 0.562)
Factor XIII	0.531 (0.464 - 0.597)	0.572 (0.532 - 0.612)	0.652 (0.600 - 0.703)	0.547 (0.524 - 0.570)
Von Willebrand factor ristocetin cofactor	0.594 (0.527 - 0.662)	0.502 (0.501 - 0.504)	0.518 (0.463 - 0.572)	0.501 (0.497 - 0.505)
post-op TRAP test AUC	0.585 (0.518 - 0.653)	0.544 (0.514 - 0.575)	0.587 (0.531 - 0.642)	0.524 (0.508 - 0.539)
post-op ASPI test AUC	0.546 (0.482 - 0.610)	0.561 (0.525 - 0.598)	0.587 (0.533 - 0.640)	0.552 (0.529 - 0.576)
post-op ADP test AUC	0.593 (0.528 - 0.657)	0.592 (0.548 - 0.637)	0.644 (0.594 - 0.695)	0.574 (0.548 - 0.600)
Haemoglobin (pre-operative)	0.683 (0.622 - 0.743)	0.538 (0.495 - 0.581)	0.533 (0.483 - 0.582)	0.546 (0.520 - 0.573)
pre-op Haematocrit	0.698 (0.639 - 0.756)	0.534 (0.500 - 0.567)	0.560 (0.509 - 0.612)	0.535 (0.513 - 0.556)
pre-op mean corpuscular volume	0.532 (0.466 - 0.597)	0.529 (0.496 - 0.561)	0.560 (0.508 - 0.612)	0.505 (0.487 - 0.523)
pre-op mean corpuscular haemoglobin	0.544 (0.478 - 0.610)	0.502 (0.482 - 0.522)	0.519 (0.466 - 0.572)	0.500 (0.488 - 0.512)
pre-op Absolute reticulocytes	0.556 (0.490 - 0.621)	0.506 (0.492 - 0.521)	0.553 (0.499 - 0.606)	0.501 (0.494 - 0.508)

Test variable	RBC_Transfusion_con_AUC	ProCoagulants_bi_AUC	ProCoagulants_con_AUC	Excessive bleeding_bi_AUC
Transferrin	0.558 (0.483 - 0.633)	0.500 (0.500 - 0.501)	0.562 (0.506 - 0.618)	0.500 (0.500 - 0.501)
Transferrin Saturation	0.583 (0.514 - 0.653)	0.516 (0.474 - 0.558)	0.520 (0.467 - 0.573)	0.511 (0.484 - 0.537)
Iron	0.607 (0.537 - 0.676)	0.507 (0.462 - 0.551)	0.507 (0.453 - 0.560)	0.507 (0.480 - 0.534)
Ferritin	0.533 (0.466 - 0.600)	0.525 (0.507 - 0.543)	0.527 (0.475 - 0.579)	0.507 (0.492 - 0.522)
pre-op lymphocyte count	0.616 (0.545 - 0.686)	0.516 (0.501 - 0.532)	0.581 (0.528 - 0.633)	0.504 (0.499 - 0.510)
pre-op monocyte count	0.515 (0.446 - 0.584)	0.501 (0.472 - 0.531)	0.554 (0.503 - 0.606)	0.507 (0.487 - 0.526)
pre-op neutrophil/lymphocyte Ratio	0.610 (0.543 - 0.676)	0.546 (0.504 - 0.589)	0.553 (0.500 - 0.607)	0.533 (0.507 - 0.560)
pre-op Platelets	0.554 (0.487 - 0.621)	0.567 (0.532 - 0.602)	0.613 (0.562 - 0.665)	0.528 (0.510 - 0.546)
pre-op IPF	0.509 (0.442 - 0.576)	0.520 (0.483 - 0.557)	0.502 (0.447 - 0.557)	0.501 (0.479 - 0.523)
pre-op MPV	0.590 (0.529 - 0.650)	0.507 (0.481 - 0.532)	0.531 (0.480 - 0.582)	0.504 (0.488 - 0.519)
Bilirubin	0.548 (0.476 - 0.620)	0.522 (0.497 - 0.547)	0.535 (0.481 - 0.589)	0.513 (0.500 - 0.526)
Albumin	0.596 (0.523 - 0.668)	0.560 (0.515 - 0.604)	0.588 (0.535 - 0.641)	0.539 (0.513 - 0.565)
Baseline eGFR	0.595 (0.530 - 0.660)	*	0.547 (0.498 - 0.595)	*
Alkaline Phosphatase	0.527 (0.454 - 0.600)	0.502 (0.490 - 0.515)	0.546 (0.487 - 0.604)	0.508 (0.499 - 0.517)
Troponin	0.585 (0.427 - 0.743)	0.562 (0.479 - 0.646)	0.556 (0.409 - 0.703)	0.522 (0.480 - 0.564)
Interleukin 6	0.586 (0.515 - 0.657)	0.503 (0.501 - 0.504)	0.518 (0.461 - 0.575)	0.501 (0.498 - 0.505)
Interleukin 8	0.592 (0.517 - 0.666)	*	0.523 (0.467 - 0.579)	*
CRP	0.563 (0.483 - 0.643)	*	0.519 (0.461 - 0.577)	*
CXCL11 (Eotaxin)	0.506 (0.432 - 0.579)	*	0.505 (0.450 - 0.560)	*
CX3CL1 (Fractalkine)	0.591 (0.521 - 0.661)	*	0.588 (0.535 - 0.641)	*
GDF-15 (mito dysfunction)	0.656 (0.590 - 0.721)	*	0.543 (0.485 - 0.601)	*
CXCL9 (iAge)	0.610 (0.544 - 0.677)	*	0.535 (0.479 - 0.592)	*
CXCL9 log transformation	0.610 (0.544 - 0.677)	*	0.535 (0.479 - 0.592)	*

eTable 5 – Diagnostic accuracy of individua

Test variable	Excessive bleeding_con_AUC	Restricted to CCB_bi_AUC	Restricted to CCB_con_AUC
post-op HB	0.620 (0.590 - 0.650)	0.501 (0.499 - 0.504)	0.521 (0.469 - 0.573)
post-op Haematocrit	0.630 (0.600 - 0.660)	0.501 (0.499 - 0.504)	0.501 (0.449 - 0.553)
post-op mean corpuscular volume	0.521 (0.487 - 0.555)	0.522 (0.496 - 0.548)	0.526 (0.476 - 0.576)
post-op mean corpuscular haemoglobin	0.535 (0.501 - 0.569)	0.519 (0.504 - 0.534)	0.514 (0.463 - 0.564)
post-op Absolute reticulocytes	0.571 (0.539 - 0.603)		0.535 (0.483 - 0.586)
post-op lymphocyte count	0.580 (0.548 - 0.612)	0.507 (0.483 - 0.531)	0.507 (0.456 - 0.558)
post-op monocyte count	0.554 (0.521 - 0.587)	0.507 (0.490 - 0.525)	0.520 (0.468 - 0.572)
post-op neutrophil/lymphocyte Ratio	0.546 (0.514 - 0.578)	0.524 (0.495 - 0.552)	0.522 (0.471 - 0.572)
post-op Platelets	0.610 (0.577 - 0.642)	0.504 (0.461 - 0.547)	0.524 (0.471 - 0.576)
post-op MPV	0.537 (0.505 - 0.570)	0.516 (0.481 - 0.550)	0.532 (0.479 - 0.585)
post-op IPF	0.500 (0.467 - 0.534)	0.571 (0.529 - 0.613)	0.563 (0.510 - 0.615)
Post-op MPV X Platelets	0.620 (0.588 - 0.653)	0.511 (0.466 - 0.556)	0.513 (0.460 - 0.567)
Post-op PT	0.588 (0.556 - 0.621)	Two cut-offs used below	0.514 (0.461 - 0.568)
Post-op PT (14s)		0.517 (0.487 - 0.547)	
Post-op PT (15s)		0.502 (0.482 - 0.522)	
Post-op APTT	0.602 (0.570 - 0.635)	0.533 (0.490 - 0.576)	0.551 (0.499 - 0.604)
Anti-Xa heparin	0.502 (0.468 - 0.536)	0.504 (0.499 - 0.509)	0.518 (0.466 - 0.570)
Endogenous thrombin potential	0.521 (0.488 - 0.554)	0.540 (0.497 - 0.583)	0.540 (0.486 - 0.594)
Clauss Fibrinogen	0.641 (0.609 - 0.673)	0.523 (0.478 - 0.568)	0.529 (0.475 - 0.583)
D dimer	0.534 (0.500 - 0.569)	0.524 (0.478 - 0.570)	0.519 (0.467 - 0.572)
Factor XIII	0.625 (0.594 - 0.655)	0.518 (0.479 - 0.557)	0.517 (0.461 - 0.572)
Von Willebrand factor ristocetin cofactor	0.519 (0.485 - 0.553)	0.503 (0.499 - 0.507)	0.545 (0.494 - 0.597)
post-op TRAP test AUC	0.567 (0.533 - 0.600)	0.524 (0.504 - 0.543)	0.533 (0.481 - 0.584)
post-op ASPI test AUC	0.571 (0.539 - 0.603)	0.511 (0.473 - 0.548)	0.509 (0.456 - 0.562)
post-op ADP test AUC	0.617 (0.585 - 0.649)	0.524 (0.482 - 0.567)	0.539 (0.487 - 0.591)
Haemoglobin (pre-operative)	0.535 (0.504 - 0.567)	0.539 (0.495 - 0.583)	0.523 (0.470 - 0.575)
pre-op Haematocrit	0.566 (0.534 - 0.599)	0.551 (0.512 - 0.589)	0.576 (0.524 - 0.627)
pre-op mean corpuscular volume	0.515 (0.483 - 0.548)	0.520 (0.493 - 0.547)	0.523 (0.472 - 0.574)
pre-op mean corpuscular haemoglobin	0.533 (0.501 - 0.566)	0.503 (0.484 - 0.522)	0.515 (0.462 - 0.567)
pre-op Absolute reticulocytes	0.534 (0.502 - 0.565)	0.500 (0.489 - 0.512)	0.554 (0.502 - 0.606)

Test variable	Excessive bleeding_con_AUC	Restricted to CCB_bi_AUC	Restricted to CCB_con_AUC
Transferrin	0.546 (0.511 - 0.581)	*	0.539 (0.485 - 0.594)
Transferrin Saturation	0.503 (0.470 - 0.536)	0.503 (0.460 - 0.547)	0.512 (0.455 - 0.569)
Iron	0.514 (0.482 - 0.547)	0.515 (0.471 - 0.559)	0.528 (0.471 - 0.585)
Ferritin	0.543 (0.510 - 0.576)	0.509 (0.484 - 0.533)	0.501 (0.445 - 0.558)
pre-op lymphocyte count	0.565 (0.533 - 0.596)	0.500 (0.491 - 0.509)	0.524 (0.472 - 0.576)
pre-op monocyte count	0.528 (0.496 - 0.560)	0.502 (0.471 - 0.533)	0.546 (0.494 - 0.597)
pre-op neutrophil/lymphocyte Ratio	0.542 (0.510 - 0.574)	0.501 (0.457 - 0.544)	0.500 (0.449 - 0.552)
pre-op Platelets	0.543 (0.511 - 0.576)	0.506 (0.477 - 0.535)	0.504 (0.452 - 0.555)
pre-op IPF	0.514 (0.483 - 0.545)	0.535 (0.497 - 0.573)	0.529 (0.476 - 0.582)
pre-op MPV	0.527 (0.496 - 0.558)	0.511 (0.483 - 0.539)	0.531 (0.478 - 0.585)
Bilirubin	0.514 (0.480 - 0.547)	0.520 (0.501 - 0.538)	0.508 (0.452 - 0.563)
Albumin	0.544 (0.510 - 0.579)	0.538 (0.498 - 0.578)	0.535 (0.480 - 0.590)
Baseline eGFR	0.516 (0.486 - 0.547)	*	0.517 (0.465 - 0.570)
Alkaline Phosphatase	0.504 (0.470 - 0.538)	0.503 (0.489 - 0.517)	0.511 (0.455 - 0.568)
Troponin	0.597 (0.511 - 0.682)	0.524 (0.446 - 0.603)	0.572 (0.409 - 0.736)
Interleukin 6	0.515 (0.479 - 0.551)	0.502 (0.498 - 0.505)	0.526 (0.471 - 0.581)
Interleukin 8	0.525 (0.490 - 0.559)	*	0.531 (0.475 - 0.587)
CRP	0.507 (0.472 - 0.542)	*	0.520 (0.463 - 0.577)
CXCL11 (Eotaxin)	0.533 (0.498 - 0.568)	*	0.546 (0.491 - 0.602)
CX3CL1 (Fractalkine)	0.544 (0.510 - 0.579)	*	0.577 (0.520 - 0.633)
GDF-15 (mito dysfunction)	0.533 (0.498 - 0.568)	*	0.514 (0.458 - 0.570)
CXCL9 (iAge)	0.533 (0.498 - 0.568)	*	0.511 (0.455 - 0.567)
CXCL9 log transformation	0.533 (0.498 - 0.568)	*	0.511 (0.455 - 0.567)

eTable 6 – Predictors, discrimination (AUROC and 95% CI), and calibration (Brier score and O:E ratio) for models to predict clinically important bleeding and its three individual components

Outcome	Model name	Predictors	AUC (95% CI)	Brier score	O:E ratio
Clinically important bleeding	TEG	TEG CK MA, TEG CK α angle,	0.611 (0.521 - 0.701)	0.154	1.003
Clinically important bleeding	ROTEM	ROTEM intem α angle,	0.588 (0.496 - 0.681)	0.154	1.002
Clinically important bleeding	Organ dysfunction biomarkers	Albumin, Haematocrit (Preop), absolute neutrophils (Preop), IPF (Preop), MPV (Preop), mean corpuscular haemoglobin (Preop), Platelets (Preop), Ferritin,	0.606 (0.512 - 0.701)	0.152	1.004
Clinically important bleeding	Ageing biomarkers	cxcl9 log2 transformation,	0.536 (0.433 - 0.639)	0.157	1.006
Clinically important bleeding	Lab tests: post-op (Sysmex + Multiplate + Coagulation)	Haematocrit (Postop), APTT, Clauss Fibrinogen, AUC ADP (Postop), D dimer, neutrophil/lymphocyte Ratio (Postop),	0.688 (0.607 - 0.769)	0.147	1.006
Clinically important bleeding	Organ dysfunction biomarkers + Ageing biomarkers + Lab tests	absolute neutrophils (Preop), APTT, Clauss Fibrinogen, Haematocrit (Preop), D dimer, MPV (Postop), Ferritin, IPF (Preop), AUC ADP (Postop), Albumin,	0.701 (0.620 - 0.781)	0.144	1.007
Clinically important bleeding	TEG + Organ dysfunction biomarkers + Ageing biomarkers + Lab tests	Ferritin, Haematocrit (Preop), absolute neutrophils (Postop), APTT, Clauss Fibrinogen, Iron, D dimer, AUC ADP (Postop),	0.694 (0.612 - 0.775)	0.145	1.008
Clinically important bleeding	ROTEM + Organ dysfunction biomarkers + Ageing biomarkers + Lab tests	Haematocrit (Preop), Clauss Fibrinogen, D dimer, Platelets (Preop), Iron, MPV (Postop), Ferritin, AUC ADP (Postop), absolute neutrophils (Preop), mean corpuscular haemoglobin (Postop),	0.692 (0.610 - 0.775)	0.145	1.008
Large volume red cell transfusion	ROTEM	ROTEM fibtem MCF, ROTEM intem α angle,	0.580 (0.392 - 0.766)	0.036	1.030
Large volume red cell transfusion	Organ dysfunction biomarkers	haemoglobin (Preop), Albumin, IPF (Preop), MPV (Preop), Platelets (Preop), Interleukin 8,	0.701 (0.501 - 0.900)	0.035	1.026
Large volume red cell transfusion	Ageing biomarkers	GDF-15, cxcl9 log2 transformation,	0.646 (0.410 - 0.880)	0.035	1.043
Large volume red cell transfusion	Lab tests: post-op (Sysmex + Multiplate + Coagulation)	Platelets (Postop), Haematocrit (Postop), absolute neutrophils (Postop),	0.745 (0.602 - 0.886)	0.034	1.041

Outcome	Model name	Predictors	AUC (95% CI)	Brier score	O:E ratio
Large volume red cell transfusion	Organ dysfunction biomarkers + Ageing biomarkers + Lab tests	absolute neutrophils (Postop), Platelets (Postop), Albumin, MPV (Preop), IPF (Preop), Haematocrit (Postop),	0.754 (0.602 - 0.903)	0.034	1.047
Large volume red cell transfusion	TEG + Organ dysfunction biomarkers + Ageing biomarkers + Lab tests	haemoglobin (Preop), Platelets (Postop), absolute neutrophils (Postop), Haematocrit (Postop), Albumin,	0.762 (0.626 - 0.897)	0.034	1.037
Large volume red cell transfusion	ROTEM + Organ dysfunction biomarkers + Ageing biomarkers + Lab tests	Albumin, absolute neutrophils (Postop), Haematocrit (Postop),	0.753 (0.608 - 0.895)	0.034	1.039
Large volume procoagulant transfusion	TEG	TEG CK MA,	0.649 (0.501 - 0.798)	0.053	1.008
Large volume procoagulant transfusion	ROTEM	ROTEM intem MCF,	0.630 (0.487 - 0.774)	0.053	1.008
Large volume procoagulant transfusion	Organ dysfunction biomarkers	mean corpuscular volume (Preop), Albumin, Platelets (Preop), Baseline eGFR, mean corpuscular haemoglobin (Preop), neutrophil/lymphocyte Ratio (Preop),	0.651 (0.497 - 0.805)	0.053	1.010
Large volume procoagulant transfusion	Lab tests: post-op (Sysmex + Multiplate + Coagulation)	AUC ADP (Postop), APTT, Clauss Fibrinogen, mean corpuscular volume (Postop), D dimer, haemoglobin (Postop), Haematocrit (Postop), neutrophil/lymphocyte Ratio (Postop),	0.720 (0.595 - 0.845)	0.052	1.020
Large volume procoagulant transfusion	Organ dysfunction biomarkers + Ageing biomarkers + Lab tests	Haematocrit (Postop), APTT, AUC ADP (Postop), Platelets (Postop), haemoglobin (Postop), Factor XIII, mean corpuscular volume (Postop), Albumin, absolute neutrophils (Postop),	0.723 (0.590 - 0.857)	0.052	1.025
Large volume procoagulant transfusion	TEG + Organ dysfunction biomarkers + Ageing biomarkers + Lab tests	Platelets (Preop), haemoglobin (Preop), AUC ADP (Postop), Albumin, absolute neutrophils (Postop), APTT, Clauss Fibrinogen, Haematocrit (Postop), haemoglobin (Postop),	0.732 (0.606 - 0.858)	0.051	1.021

Outcome	Model name	Predictors	AUC (95% CI)	Brier score	O:E ratio
Large volume procoagulant transfusion	ROTEM + Organ dysfunction biomarkers + Ageing biomarkers + Lab tests	Platelets (Preop), APTT, Clauss Fibrinogen, absolute neutrophils (Postop), Haematocrit (Postop), haemoglobin (Postop), AUC ADP (Postop), haemoglobin (Preop), Albumin,	0.732 (0.606 - 0.858)	0.051	1.021
Severe blood loss	TEG	TEG CK MA, TEG CK α angle,	0.620 (0.523 - 0.716)	0.134	1.005
Severe blood loss	ROTEM	ROTEM intem MCF,	0.606 (0.508 - 0.704)	0.135	1.003
Severe blood loss	Organ dysfunction biomarkers	Albumin, Haematocrit (Preop), absolute neutrophils (Preop), MPV (Preop), Bilirubin, Alkaline Phosphatase, IPF (Preop), mean corpuscular haemoglobin (Preop), Absolute reticulocytes (Preop), Platelets (Preop),	0.602 (0.501 - 0.702)	0.134	1.006
Severe blood loss	Ageing biomarkers	cxcl9 log2 transformation,	0.530 (0.419 - 0.641)	0.138	0.999
Severe blood loss	Lab tests: post-op (Sysmex + Multiplate + Coagulation)	AUC ADP (Postop), APTT, Clauss Fibrinogen, D dimer, haemoglobin (Postop), mean corpuscular haemoglobin (Postop),	0.680 (0.592 - 0.769)	0.130	1.007
Severe blood loss	Organ dysfunction biomarkers + Ageing biomarkers + Lab tests	Absolute reticulocytes (Postop), APTT, Clauss Fibrinogen, MPV X Platelets (Postop), Haematocrit (Preop), Ferritin, Albumin, IPF (Preop), AUC ADP (Postop), Platelets (Postop), absolute neutrophils (Preop),	0.701 (0.613 - 0.788)	0.127	1.007
Severe blood loss	TEG + Organ dysfunction biomarkers + Ageing biomarkers + Lab tests	AUC ADP (Postop), Absolute reticulocytes (Postop), Haematocrit (Preop), Ferritin, APTT, Clauss Fibrinogen, Iron, D dimer,	0.686 (0.596 - 0.776)	0.128	1.008
Severe blood loss	ROTEM + Organ dysfunction biomarkers + Ageing biomarkers + Lab tests	Iron, absolute neutrophils (Preop), Clauss Fibrinogen, D dimer, Haematocrit (Preop), Ferritin, Absolute reticulocytes (Postop), mean corpuscular haemoglobin (Postop), AUC ADP (Postop), Albumin,	0.695 (0.606 - 0.783)	0.127	1.011
