# Supplementary material for: Predictive accuracy of diagnostic tests for excessive bleeding in cardiac surgery: The COPTIC‐C study
Source: Transfusion. 2025 Oct 19;65(11):2077–90. doi: 10.1111/trf.18399 (PMC12618898; doi:10.1111/trf.18399)
Supplement: Supplementary file 2 — APPENDIX S2: Supporting information. [file TRF-65-2077-s002.docx]

**Equator checklists**

**Predictive accuracy of diagnostic tests for excessive bleeding in cardiac surgery: the COPTIC-C study**

^1^ Weiqi Liao (Senior Statistician) ORCID: 0000-0002-8605-3749

^1^ Robert Grant (Clinical Research Fellow)

^1^ Florence Y Lai (Senior Statistician)

^1^ Hardeep Aujla (Senior Research Manager)

^1^ Marcin Wozniak (Lecturer in Cardiovascular Sciences)

^1^ Hasmukh R Patel (Senior Laboratory Technician)

^2,3^ Laura Green (Professor of Haemostasis and Transfusion Medicine) ORCID: 0000-0003-4063-9768

^4^ Andrew Mumford (Professor of Haematology)

^1^ Gavin J Murphy (British Heart Foundation Chair of Cardiac Surgery, Chief Investigator)

**Affiliations**

^1^ Department of Cardiovascular Sciences, University of Leicester, UK

^2^ Blizard Institute, Queen Mary University of London, UK

^3^ NHS Blood and Transplant, London, UK

^4^ Bristol Heart Institute, University of Bristol, UK

**Corresponding Author**

Dr Weiqi Liao, Senior Statistician, Department of Cardiovascular Sciences, University of Leicester, Clinical Sciences Wing, Glenfield Hospital, Leicester LE3 9QP. Email: [weiqi.liao@leicester.ac.uk](mailto:weiqi.liao@leicester.ac.uk)

# The STARD Checklist

|  | **Section & Topic** | **No** | **Item** | **Reported on page #** |
| --- | --- | --- | --- | --- |
|  |  |  |  |  |
|  | **TITLE OR ABSTRACT** |  |  |  |
|  |  | **1** | Identification as a study of diagnostic accuracy using at least one measure of accuracy (such as sensitivity, specificity, predictive values, or AUC) | 3 |
|  | **ABSTRACT** |  |  |  |
|  |  | **2** | Structured summary of study design, methods, results, and conclusions  (for specific guidance, see STARD for Abstracts) | 3 |
|  | **INTRODUCTION** |  |  |  |
|  |  | **3** | Scientific and clinical background, including the intended use and clinical role of the index test | 4 |
|  |  | **4** | Study objectives and hypotheses | 4 |
|  | **METHODS** |  |  |  |
|  | *Study design* | **5** | Whether data collection was planned before the index test and reference standard were performed (prospective study) or after (retrospective study) | 4-5 |
|  | *Participants* | **6** | Eligibility criteria | 6 |
|  |  | **7** | On what basis potentially eligible participants were identified  (such as symptoms, results from previous tests, inclusion in registry) |  |
|  |  | **8** | Where and when potentially eligible participants were identified (setting, location and dates) | 4, 5 |
|  |  | **9** | Whether participants formed a consecutive, random or convenience series | 5, 6 |
|  | *Test methods* | **10a** | Index test, in sufficient detail to allow replication | 5 |
|  |  | **10b** | Reference standard, in sufficient detail to allow replication | 5 |
|  |  | **11** | Rationale for choosing the reference standard (if alternatives exist) | 5, eTables A-D |
|  |  | **12a** | Definition of and rationale for test positivity cut-offs or result categories  of the index test, distinguishing pre-specified from exploratory | eTables A-D |
|  |  | **12b** | Definition of and rationale for test positivity cut-offs or result categories  of the reference standard, distinguishing pre-specified from exploratory | eTables A-D |
|  |  | **13a** | Whether clinical information and reference standard results were available  to the performers/readers of the index test | 5-6, eTables A-D |
|  |  | **13b** | Whether clinical information and index test results were available  to the assessors of the reference standard | 5-6, eTables A-D |
|  | *Analysis* | **14** | Methods for estimating or comparing measures of diagnostic accuracy | 6-7 |
|  |  | **15** | How indeterminate index test or reference standard results were handled | 5, 7 |
|  |  | **16** | How missing data on the index test and reference standard were handled | 7 |
|  |  | **17** | Any analyses of variability in diagnostic accuracy, distinguishing pre-specified from exploratory | 7 |
|  |  | **18** | Intended sample size and how it was determined | 5 |
|  | **RESULTS** |  |  |  |
|  | *Participants* | **19** | Flow of participants, using a diagram | 6, Figure 1 (A) |
|  |  | **20** | Baseline demographic and clinical characteristics of participants | 8 |
|  |  | **21a** | Distribution of severity of disease in those with the target condition | 8 |
|  |  | **21b** | Distribution of alternative diagnoses in those without the target condition | 8 |
|  |  | **22** | Time interval and any clinical interventions between index test and reference standard | Figure 1 (A) |
|  | *Test results* | **23** | Cross tabulation of the index test results (or their distribution)  by the results of the reference standard | eTable 2 |
|  |  | **24** | Estimates of diagnostic accuracy and their precision (such as 95% confidence intervals) | Table 2, eTable 3, eTable 4 |
|  |  | **25** | Any adverse events from performing the index test or the reference standard | Not applicable |
|  | **DISCUSSION** |  |  |  |
|  |  | **26** | Study limitations, including sources of potential bias, statistical uncertainty, and generalisability | 11 |
|  |  | **27** | Implications for practice, including the intended use and clinical role of the index test | 11-12 |
|  | **OTHER INFORMATION** |  |  |  |
|  |  | **28** | Registration number and name of registry | 4 |
|  |  | **29** | Where the full study protocol can be accessed | 4 |
|  |  | **30** | Sources of funding and other support; role of funders | 12 |
|  |  |  |  |  |

# The TRIPOD Checklist

| **Section/Topic** | **Item** | **Checklist Item** | **Page** |
| --- | --- | --- | --- |
| **Title and abstract** | | | |
| Title | 1 | Identify the study as developing and/or validating a multivariable prediction model, the target population, and the outcome to be predicted. | 1 |
| Abstract | 2 | Provide a summary of objectives, study design, setting, participants, sample size, predictors, outcome, statistical analysis, results, and conclusions. | 2 |
| **Introduction** | | | |
| Background and objectives | 3a | Explain the medical context (including whether diagnostic or prognostic) and rationale for developing or validating the multivariable prediction model, including references to existing models. | 4 |
|  | 3b | Specify the objectives, including whether the study describes the development or validation of the model or both. | 4 |
| **Methods** | | | |
| Source of data | 4a | Describe the study design or source of data (e.g., randomized trial, cohort, or registry data), separately for the development and validation data sets, if applicable. | 4-5 |
|  | 4b | Specify the key study dates, including start of accrual; end of accrual; and, if applicable, end of follow-up. | 5 |
| Participants | 5a | Specify key elements of the study setting (e.g., primary care, secondary care, general population) including number and location of centres. | 5-6 |
|  | 5b | Describe eligibility criteria for participants. | 5, 6 |
|  | 5c | Give details of treatments received, if relevant. | N.A. |
| Outcome | 6a | Clearly define the outcome that is predicted by the prediction model, including how and when assessed. | 5-6 |
|  | 6b | Report any actions to blind assessment of the outcome to be predicted. | 6 |
| Predictors | 7a | Clearly define all predictors used in developing or validating the multivariable prediction model, including how and when they were measured. | 5-8 |
|  | 7b | Report any actions to blind assessment of predictors for the outcome and other predictors. | 6 |
| Sample size | 8 | Explain how the study size was arrived at. | 5 |
| Missing data | 9 | Describe how missing data were handled (e.g., complete-case analysis, single imputation, multiple imputation) with details of any imputation method. | 7 |
| Statistical analysis methods | 10a | Describe how predictors were handled in the analyses. | 7 |
|  | 10b | Specify type of model, all model-building procedures (including any predictor selection), and method for internal validation. | 7 |
|  | 10d | Specify all measures used to assess model performance and, if relevant, to compare multiple models. | 7-8 |
| Risk groups | 11 | Provide details on how risk groups were created, if done. | N.A |
| **Results** | | | |
| Participants | 13a | Describe the flow of participants through the study, including the number of participants with and without the outcome and, if applicable, a summary of the follow-up time. A diagram may be helpful. | 6, Figure 1 (A) |
|  | 13b | Describe the characteristics of the participants (basic demographics, clinical features, available predictors), including the number of participants with missing data for predictors and outcome. | 8, Table 1 |
| Model development | 14a | Specify the number of participants and outcome events in each analysis. | 8 |
|  | 14b | If done, report the unadjusted association between each candidate predictor and outcome. | Table 1 |
| Model specification | 15a | Present the full prediction model to allow predictions for individuals (i.e., all regression coefficients, and model intercept or baseline survival at a given time point). | eTable 6 |
|  | 15b | Explain how to the use the prediction model. | 9-10 |
| Model performance | 16 | Report performance measures (with CIs) for the prediction model. | 9-10  Table 3, eTable 6 |
| **Discussion** | | | |
| Limitations | 18 | Discuss any limitations of the study (such as nonrepresentative sample, few events per predictor, missing data). | 11 |
| Interpretation | 19b | Give an overall interpretation of the results, considering objectives, limitations, and results from similar studies, and other relevant evidence. | 10-12 |
| Implications | 20 | Discuss the potential clinical use of the model and implications for future research. | 11-12 |
| **Other information** | | | |
| Supplementary information | 21 | Provide information about the availability of supplementary resources (online supplementary materials), such as study protocol (register platform and number), Web calculator, and data sets. | Yes (notes on the left) |
| Funding | 22 | Give the source of funding and the role of the funders for the present study. | 12 |
